# An Overview of the Recent Advances in Antimicrobial Resistance

**DOI:** 10.3390/microorganisms12091920

**Published:** 2024-09-21

**Authors:** Manuela Oliveira, Wilson Antunes, Salete Mota, Áurea Madureira-Carvalho, Ricardo Jorge Dinis-Oliveira, Diana Dias da Silva

**Affiliations:** 1 Associate Laboratory i4HB—Institute for Health and Bioeconomy, University Institute of Health Sciences—CESPU, Avenida Central de Gandra 1317, 4585-116 Gandra, Portugal; aurea.carvalho@iucs.cespu.pt (Á.M.-C.); dds@ess.ipp.pt (D.D.d.S.); 2 UCIBIO—Research Unit on Applied Molecular Biosciences, Translational Toxicology Research Laboratory, University Institute of Health Sciences (1H-TOXRUN, IUCS-CESPU), Avenida Central de Gandra 1317, 4585-116 Gandra, Portugal; 3 Instituto Universitário Militar, CINAMIL, Unidade Militar Laboratorial de Defesa Biológica e Química, Avenida Doutor Alfredo Bensaúde, 4 piso, do LNM, 1849-012 Lisbon, Portugal; 4 ULSEDV—Unidade Local De Saúde De Entre Douro Vouga, Unidade de Santa Maria da Feira e Hospital S. Sebastião, Rua Dr. Cândido Pinho, 4520-211 Santa Maria da Feira, Portugal; 5 UCIBIO—Applied Molecular Biosciences Unit, Forensics and Biomedical Sciences Research Laboratory, University Institute of Health Sciences (1H-TOXRUN, IUCS-CESPU), Avenida Central de Gandra 1317, 4585-116 Gandra, Portugal; 6 Department of Public Health and Forensic Sciences and Medical Education, Faculty of Medicine, University of Porto, Alameda Prof. Hernâni Monteiro, 4200-319 Porto, Portugal; 7 FOREN—Forensic Science Experts, Avenida Dr. Mário Moutinho 33-A, 1400-136 Lisbon, Portugal; 8 REQUIMTE/LAQV, ESS, Polytechnic of Porto, Rua Dr. António Bernardino de Almeida, 4200-072 Porto, Portugal; 9 Associate Laboratory i4HB—Institute for Health and Bioeconomy, University of Porto, Rua de Jorge Viterbo Ferreira 228, 4050-313 Porto, Portugal; 10 UCIBIO—Applied Molecular Biosciences Unit, Laboratory of Toxicology, Faculty of Pharmacy, University of Porto, Rua de Jorge Viterbo Ferreira 228, 4050-313 Porto, Portugal

**Keywords:** antimicrobial resistance, antibiotic stewardship programs, epidemiology, mechanisms, novel therapeutics, One Health approach, policy interventions

## Abstract

Antimicrobial resistance (AMR), frequently considered a major global public health threat, requires a comprehensive understanding of its emergence, mechanisms, advances, and implications. AMR’s epidemiological landscape is characterized by its widespread prevalence and constantly evolving patterns, with multidrug-resistant organisms (MDROs) creating new challenges every day. The most common mechanisms underlying AMR (i.e., genetic mutations, horizontal gene transfer, and selective pressure) contribute to the emergence and dissemination of new resistant strains. Therefore, mitigation strategies (e.g., antibiotic stewardship programs—ASPs—and infection prevention and control strategies—IPCs) emphasize the importance of responsible antimicrobial use and surveillance. A One Health approach (i.e., the interconnectedness of human, animal, and environmental health) highlights the necessity for interdisciplinary collaboration and holistic strategies in combating AMR. Advancements in novel therapeutics (e.g., alternative antimicrobial agents and vaccines) offer promising avenues in addressing AMR challenges. Policy interventions at the international and national levels also promote ASPs aiming to regulate antimicrobial use. Despite all of the observed progress, AMR remains a pressing concern, demanding sustained efforts to address emerging threats and promote antimicrobial sustainability. Future research must prioritize innovative approaches and address the complex socioecological dynamics underlying AMR. This manuscript is a comprehensive resource for researchers, policymakers, and healthcare professionals seeking to navigate the complex AMR landscape and develop effective strategies for its mitigation.

## 1. Introduction

Antimicrobial resistance (AMR) is an outstanding challenge for contemporary medicine and public health, in which microorganisms adapt to withstand the effects of antimicrobial agents, rendering previous treatments ineffective [1,2]. AMR is responsible for increased morbidity, mortality, and healthcare costs, corresponding to nearly 700,000 deaths worldwide every year, which is estimated to increase to up to 10 million deaths per year by 2050. In 2014, the World Health Organization (WHO) included AMR among the top ten global health threats, a position that remains the same almost ten years later [3].

**Figure 1:**
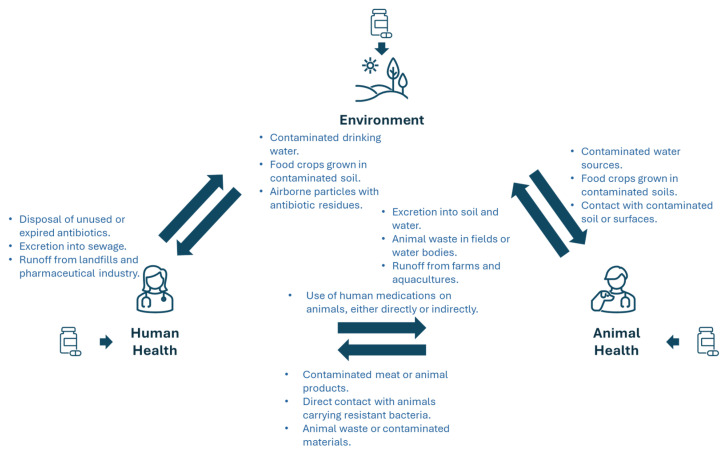
Schematic representation of antimicrobial use in agriculture and animal and human health and the flux of these drugs among all counterparts.

AMR’s emergence and dissemination is accelerated by the inappropriate use of antimicrobial agents (agriculture, veterinary and human health), poor infection control practices, inadequate sanitation, and improper food handling (Figure 1) [4,5,6]. At its core, AMR is driven by genetic changes within microbial populations, enabling them to survive and proliferate in the presence of antimicrobial drugs [7,8].

Infections caused by resistant organisms are often associated with augmented mortality, morbidity, lengths of hospitalization, and healthcare costs (i.e., an additional cost of USD 6000–30,000 when compared to those with susceptible infections or no infection) [9,10,11]. Moreover, the increase in AMR also threatens the effectiveness of medical procedures and limits the available therapies [9,12]. Therefore, addressing AMR requires a multifaceted approach comprising various sectors and stakeholders [12,13]. Effective strategies include the following:

1. Promoting ASPs to optimize the use of antimicrobial agents;
2. Implementing robust IPC strategies;
3. Investing in the research and development (R&D) of new antimicrobial agents and alternative therapies;
4. Strengthening surveillance and monitoring systems to track the emergence and dissemination of resistant pathogens.

Curiously, AMR’s appearance preceded the discovery of the first antibiotic. Salvarsan, belonging to arsphenamines, was used to treat syphilis (1910–1940s), and resistance to it emerged in the early 1920s [14]. Later, in 1928, penicillin’s introduction revolutionized medicine, offering a potent weapon against bacterial infections and saving millions of lives during the Second World War [15]. However, in less than ten years, as foreseen by Alexander Flemming himself, the extensive use of antibiotics and the selective pressure exerted led to the first reports of penicillin-resistant bacteria and, by the 1950s, the appearance of the first “hard-to-treat” infections [15,16,17]. The following decades saw rapid expansion in the discovery and development of new classes of antibiotics. However, with each new antibiotic introduced, resistance soon followed, underscoring the evolutionary arms race between microbes and medicine [18,19]. The 21st century has witnessed alarming trends in antimicrobial resistance, with the emergence of multidrug-resistant pathogens such as methicillin-resistant *Staphylococcus aureus* (MRSA), extended-spectrum β-lactamase-producing Enterobacteriaceae (ESBL), multidrug-resistant *Mycobacterium tuberculosis* (MDR-TB), and MDR *Pseudomonas aeruginosa* [20,21,22].

In agriculture, the widespread use of antibiotics for growth promotion and disease prevention significantly contributes to the emergence of resistant strains transmitted through food chains, posing risks to human health through the consumption of contaminated meat products [23,24]. Furthermore, agricultural runoff contaminates water bodies and soil with antibiotic residues, facilitating antimicrobial resistance genes’ (ARGs) dissemination among environmental bacteria [25]. Sustainable agricultural practices must prioritize judicious antimicrobial use and explore alternative strategies to mitigate the environmental dissemination of AMR (Figure 1) [26,27].

The environmental dimension of AMR accentuates its complexity and global ramifications. Resistant bacteria, genes, and antibiotics persist in diverse environmental reservoirs (e.g., soil, water, and air) that serve as hotspots for genetic exchange between microbial communities, fostering the evolution and dissemination of resistance traits. Anthropogenic activities (e.g., pharmaceutical manufacturing and improper medical waste disposal) further exacerbate environmental contamination with antimicrobial agents. Effective AMR management requires holistic strategies encompassing environmental monitoring, mitigation measures, and sustainable practices to mitigate ecological perturbations and safeguard biodiversity (Figure 1) [28,29,30].

Addressing AMR demands concerted efforts from diverse healthcare, agriculture, and environmental stakeholders [31,32]. Multidisciplinary collaboration fosters knowledge exchange, innovation, and the implementation of comprehensive strategies for AMR mitigation [31,32,33]. Surveillance systems are pivotal in monitoring resistance patterns, guiding antimicrobial stewardship programs, and informing policy interventions. Furthermore, research must prioritize developing novel antimicrobial agents, vaccines, and alternative therapies to counteract resistance mechanisms. Educational initiatives aimed at healthcare professionals, farmers, and the public are essential in raising awareness and fostering responsible antimicrobial use practices [32].

This manuscript provides a comprehensive exploration of AMR, structured into eight sections covering its development, dissemination, impact, and strategies for mitigation. Section 1 explains the genetic basis of AMR emergence in diverse microorganisms. Section 2 examines AMR’s global prevalence and distribution and elucidates the factors contributing to AMR dissemination. Section 3 debates the impact of AMR on human health, presents the strategies to address AMR, and highlights the role of surveillance programs in monitoring resistance and guiding interventions. Section 4 emphasizes the One Health approach by recognizing the interconnectedness of human, animal, and environmental health in addressing AMR. Section 5 highlights the AMR impacts on health and healthcare systems, challenges in treatment, economic costs, global implications, the need for coordinated action, and the social and ethical dimensions of this problem. Section 6 explores ASPs and IPC strategies in combating AMR, addressing responsible antibiotic use, infection prevention, global surveillance, and emerging novel approaches. Section 7 emphasizes the need for cross-sector collaboration to address AMR through the interconnectedness of human, animal, and environmental health. Section 8 reviews policies and regulations promoting responsible antimicrobial use and highlights challenges in their global implementation. Section 9: explores emerging AMR threats, the need for new treatments, and the importance of global collaboration in combating AMR. Finally, Section 10 presents the main conclusions from this work including the urgent need for a coordinated global response to AMR, integrating APS, IPC, R&D, and cross-sector collaboration to protect public health and ensure sustainable treatment solutions.

## 2. Materials and Methods

**Figure 2:**
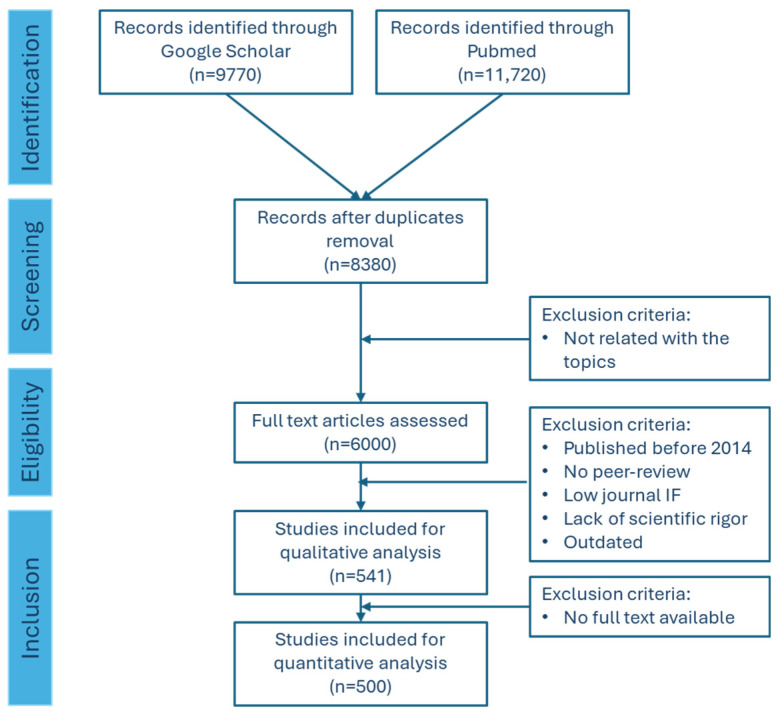
Schematic representation of the flowchart of the searched articles and the inclusion and exclusion criteria.

The manuscript selection process followed a structured approach with precise inclusion and exclusion criteria. Nearly 21,500 manuscripts were retrieved from public article databases such as PubMed (U.S. National Library of Medicine) and Google Scholar, using the keywords “Antimicrobial Resistance”, “Genetic Mechanisms”, “Epidemiology”, “Impact”, “Antibiotic Stewardship Program”, “One Health Approach”, “Policy and Regulation”, “Future Directions”, and “Challenges”. After duplicate removal, a date limit was applied (2014 and 2024). Only peer-reviewed articles with robust methods and high-quality data were considered for inclusion (journals belonging to Q1 and Q2). Manuscripts that demonstrated practical relevance, showing clear applications to casework scenarios, were also included. As exclusion criteria, manuscripts that did not directly address the core topics, lacked scientific rigor, or were outdated were removed from the analysis. The research team analyzed more than 500 manuscripts at the end of this selection process. This process ensured that the revised manuscript incorporated the most pertinent and high-quality research, providing a comprehensive update on AMR (Figure 2).

## 3. Mechanisms of Antimicrobial Resistance

### 3.1. Development of Antimicrobial Resistance

AMR, a significant global health challenge, poses a threat to the effectiveness of drugs that combat microorganisms (i.e., bacteria, viruses, fungi, and parasites), which have developed diverse biological mechanisms to evade the effects of antimicrobial agents (Table 1).

#### 3.1.1. Antimicrobial Resistance Mechanisms: Bacteria

In the AMR context, bacteria are some the most extensively studied microorganisms, mainly due to their capacity to develop several mechanisms that enable them to survive antibiotic exposure. In one of these mechanisms, known as target modification, bacteria alter their molecular targets for antibiotics, preventing the drugs from effectively binding [34]. For instance, mutations in bacterial ribosomal RNA (rRNA) hinder the binding of aminoglycosides, enabling the bacteria to continue protein synthesis despite the antibiotic’s presence [35]. The recent study by Babosan and co-workers demonstrated the importance of nonessential transfer RNA (tRNA) and rRNA modifications in the bacterial response to antibiotic stress, while it proposes that differential translation and codon decoding play an essential role in the stress response [36].

In some bacteria, efflux pumps actively transport a broad range of drugs outside the cytoplasm, reducing their intracellular concentrations and allowing the bacteria to survive at higher antibiotic concentrations [37,38]. These membrane proteins are particularly significant in antibiotic resistance to tetracyclines, fluoroquinolones, and macrolides [39].

Bacteria produce enzymes, often involved in the biosynthesis of the cell wall, nucleic acids, and metabolites, which serve as a direct target for antibiotics. Structural changes in these enzymes also change the bacteria’s resistance profiles [40]. For instance, β-lactamases hydrolyze the β-lactam ring in penicillin, cephalosporin, and carbapenems (relatively resistant to β-lactamase), making these antibiotics ineffective [41,42]. Therefore, β-lactam antibiotics are frequently used in association with beta-lactamase inhibitors [41,43]. Nevertheless, continuous bacterial evolution and mutation lead to the appearance of new β-lactamases that can resist these inhibitors [42].

Some bacteria, especially Gram-negative ones, possess a mechanism that reduces the cell membrane permeability, preventing antibiotics from entering the cell. Therefore, the bacterial outer membrane acts as a selective diffusion barrier, limiting antibiotic influx [44] by two main pathways: diffusion using protein channels (hydrophilic antibiotics) and a lipid-mediated pathway (hydrophobic antibiotics). Modifications in the lipid and protein compositions of the outer membrane can significantly impact the bacterial sensitivity to antibiotics [45,46]. Better knowledge of the molecular basis of outer membrane permeability and the physicochemical parameters influencing antibiotic translocation through porins is essential in combating antibiotic resistance [44].

Finally, some bacterial strains form biofilms, becoming highly resistant to antibiotics due to the presence of a self-produced protective extracellular matrix that limits drug influx, while the bacteria within, often presenting lower metabolic activity and longer doubling rates, are less susceptible to antibiotics targeting actively dividing cells [47,48]. The biofilm structure presents two advantages concerning bacterial survival: it creates gradients of nutrients and oxygen, leading to diverse metabolic states and promoting antibiotic tolerance, while facilitating HGT and inducing higher mutation frequencies [49]. These mechanisms provide critical knowledge for the development of new strategies to combat biofilm-associated infections and address antibiotic resistance [49,50].

#### 3.1.2. Antimicrobial Resistance Mechanisms: Viruses

Antiviral resistance arises through mechanisms that are distinct from those in bacteria. These differences are because viruses rely on host cells for replication and do not possess the same cellular targets as bacteria [51,52].

Traditional therapeutical approaches use antiviral drugs targeting viral enzymes (e.g., reverse transcriptase, protease, or RNA polymerase). Mutations in the genes encoding these enzymes reduce the drug’s binding affinity, allowing the virus to continue replicating. For instance, HIV resistance is due to reverse transcriptase and protease inhibitors [53]. To solve this problem, new targets based on components that are essential for the virus but not for the host (e.g., small interfering RNA (siRNA), CRISPR, and small-molecule inhibitors targeting cellular kinases) are being studied and present promising results [54]. These new therapeutical developments also include proteolysis-targeting chimeras (PROTACs), which leverage the ubiquitin–proteasome system for protein degradation. Compounds targeting host factors (e.g., human dihydroorotate dehydrogenase) have also shown good results as potential broad-spectrum antivirals [76].

Some antivirals block viral entry into host cells by targeting viral surface proteins. Mutations in these proteins can prevent the drug from binding, allowing the virus to infect cells despite treatment. This mechanism is relevant in resistance to entry inhibitors used in treating HIV [77].

Finally, some antiviral drugs require activation by viral or host enzymes to become effective [54]. Therefore, mutations that reduce the efficiency of this activation result in resistance to these drugs. For instance, acyclovir resistance in herpes simplex virus (HSV) is due to mutations in the viral thymidine kinase gene (*TK*), resulting in complete activity loss, reduced expression, or altered affinity [78].

#### 3.1.3. Antimicrobial Resistance Mechanisms: Fungi

Fungal pathogens develop resistance to antifungal agents through mechanisms analogous to those in bacteria and viruses but are characterized by unique aspects associated with their specific biology [79]. The emergence of multidrug-resistant fungi (e.g., *Candida auris* and *Aspergillus* spp.) has significantly complicated the treatment options [80]. Better knowledge of these resistance mechanisms would assist in developing alternative therapeutic strategies to combat refractory mycoses [80,81].

Fungi can alter the targets of antifungal drugs. For instance, mutations in the lanosterol 14-alpha-demethylase gene (*ERG11*), the target for antifungals, result in several aminoacidic changes that change the drug’s capacity to bind and inhibit the gene, resulting in decreased susceptibility to azoles (fluconazole, itraconazole, and voriconazole) [55].

Fungi also possess efflux pumps, mainly belonging to the ATP-binding cassette (ABC) or major facilitator superfamily (MFS), that expel antifungal drugs from fungal cells [56,57]. The overexpression of these pumps is a common resistance mechanism to azoles and other antifungals in *Candida* species and other fungi [58,59,60]. Efflux-mediated resistance can be surpassed by alternative antifungals that are not pump substrates, target transcriptional regulators and stress response pathways, block the energy supply, and directly inhibit these pumps [61].

Fungi can modify their cell wall compositions to reduce the binding or penetration of antifungal agents. For instance, resistance to echinocandins primarily arises from mutations in the *FKS* gene, which encodes the catalytic subunit of glucan synthase and is associated with reduced sensitivity to the enzyme [62]. By modifying echinocandin structures with some dehydroxylated derivatives, it is possible to surpass this resistance mechanism [63].

Fungi can also form biofilms, which are particularly problematic in infections involving medical devices (e.g., catheters, pacemakers, and prosthetic joints) [64]. Due to their structure—mainly the extracellular matrix—biofilms are intrinsically resistant to conventional antifungal therapeutics [65]. Developing novel strategies to prevent and treat biofilm formation is crucial in managing these difficult-to-treat infections [66].

#### 3.1.4. Antimicrobial Resistance Mechanisms: Parasites

Parasites (e.g., protozoa and helminths) are becoming more resistant to antiparasitic drugs [82,83]. Resistance is induced by frequent treatment, underdosing, non-compliance with the duration of the dosing regimen, poor drug quality, drug interactions, poor or erratic absorption and misdiagnosis, and parasite genetics [82]. Several mechanisms for antiparasitic drug resistance have been proposed [82].

Parasites can accumulate mutations in the genes encoding the molecular targets of antiparasitic drugs, leading to reduced drug efficacy. The genes involved differ from species to species, and the same species can exhibit the involvement of several genes. For instance, in *Plasmodium falciparum* (malaria parasites), antifolate drug resistance involves point mutations in dihydropteroate synthase (*dhps*) and dihydrofolate reductase (*dhfr*) [84]; mutations in the Kelch13 protein (*K13*), involved in multiple intracellular processes including hemoglobin catabolism, are associated with artemisinin resistance [67,68]; and overexpression or mutations in the digestive vacuole membrane-bound ABC transporter *PfMDR1* are associated with resistance to chloroquine and other 4-aminoquinolines [69,70]. In *Toxoplasma gondii* (toxoplasmosis), resistance to auranofin is associated with mutations in redox-relevant genes, enhancing the parasite’s ability to manage oxidative stress [71].

Another mechanism is associated with the efflux pumps that actively remove antiparasitic drugs from their cells. This mechanism has been observed in *P. falciparum*, where these pumps enable the efflux of drugs (e.g., chloroquine, amodiaquine, and piperaquine) from the digestive vacuole, blocking their antimalarial action [70].

Parasites can alter their metabolic pathways to bypass the effects of drugs. For instance, mutations in the genes encoding enzymes in the glycolytic pathway confer resistance to drugs targeting this pathway in trypanosomes (sleeping sickness) [72].

Finally, some antiparasitic drugs require activation within the parasite to exhibit a therapeutical effect—a mechanism known as a “magic bomb” [73]. Modifications in this activation process can lead to resistance. For instance, nifurtimox and benznidazole, often used to control *Trypanosoma cruzi* (Chagas disease), are activated by a mitochondrial nitroreductase (*NTR*) that, when downregulated, results in cross-resistance [74]. Artemisinin-based drugs for malaria require heme-mediated activation, and resistance is associated with changes in the parasite’s life cycle and enhanced stress responses [75].

### 3.2. Genetic Mechanisms of Antimicrobial Resistance

The development of AMR is mediated by several genetic mechanisms that enable microorganisms to adapt and survive in the presence of these agents [85,86]. These mechanisms will be covered in the following sections.

#### 3.2.1. Mutations

Mutations, a fundamental genetic process, introduce random changes in an organism’s DNA sequence. These can occur spontaneously or be induced by external factors, such as antimicrobial exposure [87]. Spontaneous mutations naturally occur during DNA replication, resulting in the emergence of resistant strains if the mutation affects a gene involved in drug susceptibility [88]. Despite the action of replication error repair systems, some errors escape repair due to temporal constraints [89]. For example, a point mutation in the gene encoding bacterial DNA gyrase confers resistance to quinolones by reducing the drug’s binding affinity [90]. The rate of spontaneous mutation during replication varies among microorganisms and even between those that are closely related. For instance, herpes simplex virus type 2 (HSV-2) presents a higher mutation rate than HSV-1 (laboratory strains: 9–16-fold; clinical isolates: nearly 30-fold) [91]. However, the emergence of resistance often results from mutations in several genes. For instance, in *M. tuberculosis*, resistance to PA-824 is associated with mutations in genes related to prodrug activation and the F420 biosynthetic pathway (genes: *ddn*, *fgd1*, *fbiA*, *fbiB*, and *fbiC*), resulting in reduced sensitivity [92].

Exposure to sub-lethal concentrations of antimicrobial agents induces DNA damage and genomic instability, leading to a higher mutation rate in microorganisms and, potentially, the emergence of new AMR—antibiotic-induced mutagenesis [93,94]. This phenomenon occurs due to the production of reactive oxygen species (ROS) and the consequent oxidative stress, which induces error-prone polymerases, misbalancing nucleotide metabolism or directly affecting DNA [95]. As a result, a wide range of genetic perturbations can be observed, from single-nucleotide polymorphisms (SNPs) to severe chromosomal rearrangements [94]. It has been suggested that heavy metals also increase the mutation rates and allow the emergence of mutants with resistance to multiple antibiotics, highlighting the potential role of environmental contaminants in promoting antibiotic resistance [96]. Besides these two, other factors may contribute to the increased mutation rate in bacteria and the potential emergence of antimicrobial resistance, including chemical pollutants (e.g., biocides, nanoparticles, and disinfection byproducts) and non-chemical stressors (e.g., ultraviolet radiation, electrical stimulation, and starvation) [97].

#### 3.2.2. Horizontal Gene Transfer

Horizontal gene transfer (HGT), an essential process for the evolution of various organisms, allows for genetic material to be transferred between distinct species, bypassing traditional modes of inheritance [98,99]. This process contributes to genome plasticity and adaptive evolution in prokaryotes and eukaryotes by introducing new genes and functions or serving as a source of phenotypic innovation and niche adaptation [100,101]. HGT has been implicated in the emergence and dissemination of pathogenic traits in eukaryotes, such as antibiotic resistance and virulence factors [102,103]. There are three classical modes of HGT: conjugation, transformation, and transduction. Recently, a fourth mode of DNA transfer through extracellular vesicles—vesiduction—has been proposed [104].

Conjugation involves the unidirectional transfer of genetic material between a donor and a recipient bacterium through direct cell-to-cell contact [105]. Despite small differences in Gram-negative and -positive bacteria, the transfer involves conjugative plasmids and integrating conjugative elements (ICEs, including transposons) that resort to complex machinery that includes a type IV secretion system (T4SS), relaxosome, and pilus [106,107]. This process is regulated by plasmid- and host-encoded factors responding to environmental and physiological signals [108,109]. For instance, in *Bacillus subtilis*, the conjugative plasmid pLS20 uses several control levels (e.g., a quorum-sensing system and strict repression of the main conjugation promoter Pc), ensuring that conjugation genes are only expressed under favorable conditions [110]. However, phage plasmids, which carry numerous clinically important resistance genes usually included in integrons, achieve resistance through infection and lysogenic conversion, potentially transferring genes to distant bacteria without cell-to-cell contact [111]. Plasmids often carry multiple resistance genes, enabling the recipient bacterium to simultaneously acquire resistance to several antibiotics in a single conjugation event [112]. Conjugative plasmids have developed mechanisms to disable host defenses and compete with other plasmids, facilitating their transmission and establishment in new bacterial hosts [113]. The *mecA* gene, responsible for the antibiotic resistance observed in MRSA, encodes for penicillin-binding protein 2a (PBP2a) with low affinity for β-lactam antibiotics (e.g., methicillin and other penicillin derivatives). Normally, β-lactam antibiotics bind to penicillin-binding proteins (PBPs) and inhibit bacterial cell wall synthesis. In MRSA, PBP2a’s altered structure allows the cell wall to be biosynthesized, even in the presence of these antibiotics, rendering them ineffective. The gene is located on a mobile genetic element known as the staphylococcal cassette chromosome mec (SCCmec), which can be horizontally transferred between *Staphylococcus* species, further disseminating resistance [114]. Carbapenemases, enzymes produced by Gram-negative bacteria, are responsible for carbapenem resistance. These enzymes belong to different classes, with the most notable being the *Klebsiella pneumoniae* carbapenemase (KPC), New Delhi metallo-beta-lactamase (NDM), Verona integron-encoded metallo-beta-lactamase (VIM), and oxacillinase (OXA). Carbapenemases break down the β-lactam rings of carbapenems, preventing PBPs’ inhibition and neutralizing their antibacterial effects. Plasmids encoding carbapenemases, such as *KPC*, *NDM*, and *OXA-48-like*, found in Enterobacteriaceae (CRE), *Acinetobacter*, and *Pseudomonas*, facilitate the rapid dissemination of carbapenem resistance [115]. Plasmids carrying aminoglycoside-modifying enzyme (*AME*) genes, such as *aac(6′)-Ib* and *aph(3′)-VI*, which often co-occur with other resistance genes, enable bacteria to resist aminoglycosides and β-lactams [116]. The co-occurrence of AME and carbapenemase genes, such as *blaNDM-1* and *blaKPC-2*, observed in Enterobacterales, further limits the available treatment options [117]. Colistin resistance, due to plasmids with *mcr* genes (*mcr-1* and *mcr-10*) in *E. coli* and other species, further complicates the treatment of infections caused by multidrug-resistant Gram-negative bacteria, especially in co-occurrence with other antibiotic resistance genes [118]. Plasmids carrying extended-spectrum β-lactamase (ESBL) genes, such as *TEM*, *SHV*, and *CTX-M*, are frequently found in bacteria such as *E. coli* and *K. pneumoniae* and hydrolyze β-lactam antibiotics and often carry additional genes, resulting in multidrug resistance to aminoglycosides and fluoroquinolones [119]. It has been proven that when several bacterial species are present in co-cultures, interspecies plasmid transfer may occur, facilitating ARGs’ dissemination among bacteria and allowing the emergence of multidrug-resistant bacteria [120].

Natural transformation, where bacteria uptake and incorporate environmental DNA, is an important mechanism for AMR acquisition, influenced by biotic and abiotic factors, (e.g., environmental stresses and antimicrobials’ presence) [121,122]. This process facilitates the transfer of large antibiotic resistance islands between bacterial species, particularly in pathogens such as *Acinetobacter baumannii* [123]. Moreover, bacteria can incorporate these mobile genetic elements carrying ARGs and then remove them, better adjusting to intermittent stress exposure and maximizing their fitness [124].

Transduction involves phages (bacteriophages) that carry bacterial DNA, including ARG and virulence genes, between bacterial hosts [125]. This process plays an important role in acquiring, maintaining, and disseminating antibiotic resistance [126]. For instance, it contributes to the dissemination of resistance genes among foodborne pathogens, especially in Enterobacteriaceae [127]. Transduction can occur through three different mechanisms: generalized (i.e., phages package chromosomal and/or bacterial DNA and transfer it to another bacterium), specialized (i.e., limited to the transfer of specific sets of genes when viral and bacterial host DNA are encapsidated as a hybrid molecule), and lateral transduction (i.e., a natural part of the phage life cycle that transfers bacterial chromosomal DNA at frequencies at least 1000-fold greater than previous mechanisms) [128].

Vesiduction, a HGT mechanism that involves membrane vesicles (MVs), has been recently proposed as a fourth mode of DNA transfer [104]. MVs—particularly outer membrane vesicles (OMVs) in Gram-negative bacteria—play important roles in bacterial communication, secretion, and self-defense, including protection against antibiotics [129]. For instance, in *Enterococcus faecium*, vancomycin exposure induces the increased production of MVs containing *vanA* and *vanB* resistance genes [130].

#### 3.2.3. Selection Pressure and Its Role in AMR Development

Selection pressure is the environmental force that drives AMR evolution. When a given microorganism is exposed to an antimicrobial agent, those with mutations conferring either resistance or acquired resistance genes have a survival advantage and proliferate; those that are susceptible are eliminated, leading to the dominance of resistant strains within the population [131]. The environment acts as an AMR reservoir, with hospital wastewater and agricultural runoff contributing to the dissemination of resistant microorganisms and its ARGs in the soil, water, and even air [29]. Diverse factors (e.g., infection control standards, sanitation, and travel) affect the role of selection pressure in AMR development, being essential to understand the ecological and evolutionary processes leading to ARGs’ mobilization and dissemination in order to prevent it [132].

##### Antimicrobial Use

Antimicrobials’ widespread and often inappropriate use in healthcare, agriculture, and animal husbandry creates strong selection pressure for the development and dissemination of resistance [133]. Agriculture, including livestock and aquaculture, significantly contributes to AMR development due to extensive antimicrobial use, often at subtherapeutic levels [134,135], inducing strong selection pressure for resistant bacteria, which can potentially be transmitted to humans through the food chain, direct animal contact, and environmental contamination [133]. This problem is exacerbated by the large, interconnected human population and the frequent misuse of antimicrobials in healthcare [136]. Addressing AMR requires an integrated and multifaced approach, comprising improved surveillance, reduced antimicrobial usage in agriculture and healthcare, the development of new drugs, and education programs to change public perceptions of antimicrobial use [133,136].

##### Suboptimal Dosing

Inadequate antibiotic dosing or incomplete courses are also responsible for AMR emergence, and resistant infections often occur during or after antibiotic therapy [137]. Protocols developed to optimize pharmacokinetic/pharmacodynamic principles in antibiotic dosing maximize their efficacy and suppress resistance [138]. On the other hand, antibiotic exposure also induces hypervirulence in pathogens, potentially worsening disease outcomes [139]. ASPs promote prudent antimicrobial use, including appropriate diagnostics and IPC strategies, limiting AMR emergence in clinical settings by applying interventions such as guidelines, pre-authorization, reviews, audits, feedback, and education [140].

##### Environmental Factors

Water, soil, and sediments act as reservoirs and vectors for resistant microorganisms and ARGs [29]. Antibiotic residues disseminated in the environment, particularly near intensive animal husbandry farms, create selective pressure for resistant organisms at concentrations below the minimum inhibitory concentration [141]. Moreover, anthropogenic activities (e.g., hospital and municipal discharges) disseminate resistant bacteria and their genes in aquatic environments [142]. To address this issue, further research is required, focusing on the relationship between environmental antibiotic concentrations and resistance prevalence, improved wastewater treatment, and management practices [143,144].

### 3.3. Interplay between Genetic Mechanisms and Selection Pressure

The interplay between genetic mechanisms and selection pressure in AMR is complex and multifaceted. On the one hand, bacteria adapt to host defense systems and antimicrobial agents through diverse mechanisms (e.g., structural changes, enzymatic processes, and gene regulation) [145]. On the other hand, mobile genetic elements (e.g., plasmids and transposons) disseminate ARGs, and non-genetic factors (e.g., transcriptional heterogeneity) facilitate the development of genetic drug resistance [146]. Additionally, epigenetic modifications (e.g., DNA methylation and non-coding RNA regulation) contribute to bacterial adaptation and antibiotic resistance [147]. Finally, rapid AMR dissemination is further intensified by processes of genetic recombination and selective evolutionary pressure induced by the inappropriate use of antimicrobial drugs [148].

Recently, bioinformatics approaches, including whole-genome sequencing (WGS) and other high-throughput technologies, have become valuable tools for the study of AMR mechanisms and the discovery of new resistance signatures [149].

### 3.4. Clinical and Public Health Implications

In low- and middle-income countries (LMICs), AMR is a major public health concern [150]. Understanding the mechanisms of AMR is essential in developing effective strategies to combat this global health threat. For instance, the ESKAPE pathogens (*E. faecium*, *S. aureus*, *K. pneumoniae*, *A. baumannii*, *P. aeruginosa*, and *Enterobacter* species), along with *Clostridioides difficile*, are responsible for most nosocomial infections worldwide. This not only demonstrates the challenges posed by multidrug-resistant organisms but also highlights the importance of understanding their specific resistance mechanisms for the development of novel antimicrobial agents [151,152,153].

To reduce AMR emergence, clinical practitioners should use antibiotics judiciously. Nearly 50% of antibiotic prescriptions are considered unnecessary or inadequate (e.g., overprescribing and switching to same-class agents for therapy failure) [154] due to factors such as uncertain diagnosis and patient influences [152]. To address the AMR crisis, clinicians must implement adequate strategies, such as rapid microbiological diagnostics, the use of inflammation markers, shorter antibiotic courses, and individualized therapies [153]. Healthcare institutions should develop and enforce guidelines for appropriate antimicrobial use [152]. The U.S. CDC’s 2019 Antibiotic Resistance Threats Report provides valuable insights for frontline providers on the national AMR crisis [154].

Public health initiatives, such as education and awareness campaigns, should focus on improving the use of antimicrobial drugs, both in human and animal health [155]. Moreover, implementing effective IPC strategies, such as improved hygiene, sanitation, and facility decontamination, is critical to prevent the dissemination of resistant microbes [150]. Complementary surveillance programs have been developed to monitor AMR trends and guide interventions to reduce AMR dissemination [156]. The One Health approach, which recognizes the interconnectedness of human, animal, and environmental health, addresses AMR comprehensively [157].

Novel and innovative vaccine technologies (e.g., reverse vaccinology) and adjuvants have been proven effective in targeting multidrug-resistant bacteria [158], with their wider application being limited only by the need to better understand immunological mechanisms and identify correlates of protection [159]. Moreover, new approaches for antibiotic delivery and alternative modalities, such as targeting host factors and blocking bacterial virulence, are also being explored [160]. Rapid, accurate, point-of-care diagnostics not only allow for improved prescribing practices but also enable more efficient clinical trials [161].

## 4. Epidemiology of Antimicrobial Resistance

AMR affects human and veterinary medicine and threatens to reverse decades of progress in the treatment of infectious diseases [162,163]. Therefore, population-based studies are required to understand the burden, distribution, and determinants of AMR, as well as to develop effective prevention and management strategies [164]. These studies will provide unbiased prevalence estimates and overcome the limitations of laboratory-based surveillance (e.g., weak laboratory capacity, poor health systems, and limited resources) common in LMICs [165,166]. To address this global challenge, the WHO has developed action plans to promote new antibiotics, reduce antibiotic misuse, and improve surveillance [167]. The metagenomic analysis of urban sewage has been proposed as an economically feasible approach for continuous global AMR surveillance [168].

### 4.1. Global Prevalence and Distribution of Antimicrobial Resistance

**Figure 3:**
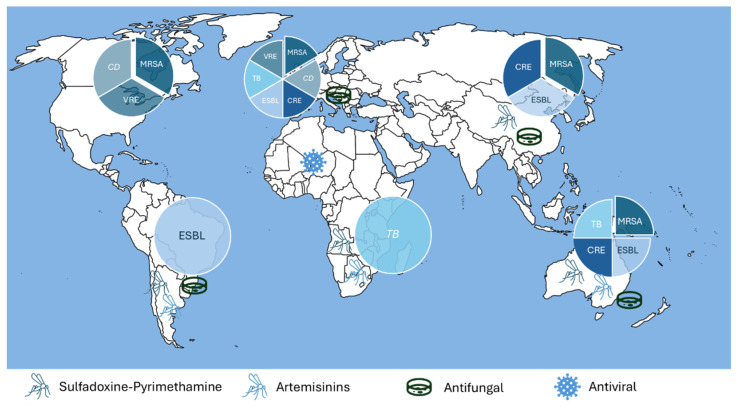
Global distribution of the main multi-resistant strains and antimicrobial resistance (*CD*: *Clostridium difficile*; CRE: carbapenem-resistant Enterobacterales; ESBL: extended-spectrum beta-lactamase; MRSA: methicillin-resistant *Staphylococcus aureus*; VRE: vancomycin-resistant *Enterococcus*; TB: multidrug-resistant *Mycobacterium tuberculosis*).

AMR’s global distribution significantly varies across worldwide locations (Figure 3) [168]. Multidrug-resistant organisms (MDROs) (e.g., MRSA; vancomycin-resistant Enterococci—VRE; CRE; hospital-associated *C. difficile* infection—HA-CDI; and community-associated *C. difficile* infection—CA-CDI) have become gradually more common in healthcare settings and the community [169,170,171].

MRSA is globally widespread, particularly in North America, Europe, and East Asia, mainly due to acquiring virulence factors and resistance to multiple antibiotics [172]. It has been proposed that international travel has contributed to MRSA dissemination across continents, potentially replacing the endemic strains with these more transmissible variants [173].

The SENTRY Antimicrobial Surveillance Program reported that VRE is responsible for more than 8% of enterococcal isolates globally, with North America having the highest prevalence at 21.6% [174].

CRE presents a critical issue in healthcare settings, especially in South and Southeast Asia, where prolonged hospital stays are associated with high mortality rates, mainly due to limited access to effective treatments [175]. The frequent treatment failures and the increased economic burden on patients and nations has forced the WHO to prioritize CRE as a top pathogen of public health importance to contain its dissemination [176].

Another important emerging MDRO is *C. difficile*, a nosocomial pathogen with a global distribution, primarily affecting individuals with disrupted gut microbiota due to antibiotic use [177]. Epidemiological data indicate its presence in various environmental reservoirs, including healthcare settings, where its transmission is facilitated [178]. The emergence of hypervirulent strains like ribotype 027 has exacerbated this trend, driving higher morbidity and mortality rates worldwide [178].

Viral AMR is also a growing concern since several viruses (e.g., HIV, hepatitis B and C, influenza, herpes simplex, varicella-zoster) have become resistant to antiviral therapies [179,180,181]. This is particularly problematic in regions such as sub-Saharan Africa, where the expansion of antiretroviral therapy (ART) is associated with poor staff capacities and infrastructure to support consistent treatment adherence, leading to the emergence of resistant strains [182,183].

Although less discussed, fungal AMR also poses a significant threat to immunocompromised patients, mainly those with hematologic malignancies and organ transplants [184,185]. Some *C. auris* strains are resistant to multiple antifungal agents (e.g., azoles, amphotericin B, and echinocandins) [186]. This pathogen, first identified in Japan in 2009, has rapidly disseminated across Asia, Europe, and the Americas [187]. Its ability to persist on surfaces, colonize patients for extended periods, and be efficiently transmitted from person to person makes this pathogen difficult to control [188], being often associated with high mortality rates [189]. Azole resistance in *Aspergillus* species, mainly *A. fumigatus*, is a growing concern worldwide, and the transboundary spread of this microorganism in many countries has been suggested [190].

AMR in parasites, notably in malaria-causing *P. falciparum*, is also a major concern, significantly threatening efforts to control and eliminate this disease [191]. Resistance to artemisinins has been disseminated from Southeast Asia, in the Greater Mekong Subregion, to Africa and South America [192]. Chloroquine resistance, first reported in South America and Southeast Asia (early 1960s), was disseminated to East Africa (late 1970s) and then westward [193]. Finally, the emergence and dissemination of resistance to sulfadoxine–pyrimethamine occurred in Southeast Asia and South America, subsequently spreading to Africa, similar to that observed for chloroquine [194].

### 4.2. Trends in Antimicrobial Resistance over Time and across Different Regions

Although with significant regional variations, AMR’s prevalence is steadily increasing worldwide. Monitoring data from various surveillance networks (e.g., European Antimicrobial Resistance Surveillance Network, EARS-Net) reveal that the incidence of resistant infections has significantly risen over recent decades [195]. For instance, while the prevalence of MRSA has decreased in some European countries due to rigorous IPC strategies, it remains high in others [196]. Moreover, there has been an alarming rise in resistance to last-resort antibiotics, such as colistin, especially in Gram-negative bacteria [197].

The distribution of AMR is heavily influenced by regional factors, including the misuse of antibiotics, inadequate living conditions, poor healthcare infrastructure, and deficient public health policies [198]. For instance, in many African countries, weak healthcare systems, limited access to proper diagnosis, and non-prescription antibiotic dispensing have contributed to high levels of AMR [199,200]. Resistant infections, such as those caused by *Salmonella typhi* and *K. pneumoniae*, are particularly prevalent, complicating treatment protocols. International initiatives, such as the Global Antimicrobial Resistance Surveillance System (GLASS; WHO) and AMRSNET (Africa CDC), aim to enhance AMR surveillance and control in this continent [200].

South and Southeast Asia are recognized as hotspots for AMR, mainly driven by the high usage of antibiotics in healthcare and agriculture [201]. This region has experienced rapid economic growth associated with increased animal product consumption, contributing to AMR emergence [202]. Antibiotic use for growth promotion and treatment is prevalent, with a significant overlap between critically important antibiotics used in animals and humans [203]. Easy access to antibiotics, coupled with limited veterinary care, drives inappropriate use [203]. While some countries have policies to address AMR, most lack detailed implementation plans [204]. Developing locally relevant strategies based on situation analysis is critical, as one-size-fits-all policies may be inappropriate for the region’s unique context [204]. In contrast, Western European countries have reduced their MRSA rates by implementing stringent IPC strategies (e.g., targeted screening, decolonization, hand hygiene, and ASPs). However, this region continues to face challenges with Gram-negative bacteria, such as CRE, especially in refugee patients [205,206,207]. On the other hand, countries from Eastern Europe continue to struggle with high levels of AMR, mainly due to overuse and weaker healthcare systems. It has been reported that the drivers of irrational antibiotic use in some of these countries include a lack of public awareness, access to antibiotics without a prescription, and inadequate medical training [208]. For instance, Poland has one of the highest rates of antibiotic consumption among all European countries [209].

The AMR trends have fluctuated in the United States, with recent increases in resistant gonorrhea and CRE. While the MDR gonorrhea rates have remained relatively low over the past three decades, a peak was observed in 2011 and it settled at lower values five years later [210]; in recent years, there has been a concerning increase in azithromycin resistance [211]. Concerning CRE, the rates slightly increased between 2015 and 2019 [212], with the highest infection rates observed in long-term acute-care hospitals [213]. Latin America is experiencing an emerging AMR problem, with increasing reports of resistant infections associated with healthcare settings, such as carbapenem resistance in *A. baumannii* and *P. aeruginosa* [214]. Healthcare professionals recognize the importance of antibiotic stewardship; however, they do not perceive AMR as a problem in their facilities [215]. Furthermore, rural settings also contribute to AMR through intensive animal production and agricultural practices [216].

In viral infections, the widespread use of ART has led to a significant decline in HIV-related deaths globally, avoiding a total of 9.5 million deaths between 1995 and 2015 [217]. However, the emergence of drug-resistant HIV strains is beginning to undermine these positive results, threatening the effectiveness of ART and the control of the HIV epidemic [218]. These alarming results are mainly due to non-compliance, insufficient dosages, drug interactions, and viral mutations [218]. Similarly, resistance to influenza antivirals, such as oseltamivir, has been documented, with its prevalence varying across different influenza seasons [219].

### 4.3. Factors Contributing to the Dissemination of Antimicrobial Resistance

AMR dissemination, regulated by an interconnected network of factors, includes healthcare practices, antimicrobial use in agriculture, international travel, medical tourism, and environmental factors.

In healthcare settings, the inadequate use of antibiotics is the main contributor to AMR, being commonly influenced by other factors such as a lack of public knowledge, access to antibiotics without a prescription, inadequate medical training, and insufficient diagnostic tests [208]. In many regions, antibiotics are frequently overprescribed, often for conditions where they are ineffective, such as viral infections. For instance, in the United States, at least 30% of outpatient antibiotic prescriptions are considered unnecessary, rising to 50% for respiratory tract infections [220]. This overuse contributes to antimicrobial resistance, adverse drug events, and increased healthcare costs. Additionally, inadequate infection control practices in hospitals contribute to the dissemination of resistant organisms, mainly Gram-negative bacteria [221]. For instance, the overuse of broad-spectrum antibiotics in intensive care units (ICUs) has been linked to the emergence of resistant strains such as CRE [222].

Healthcare-associated infections (HAIs) caused by resistant bacteria (e.g., MRSA, VRE, the “ESKAPE” group, and HA-CDI) are particularly problematic in hospitals, where they rapidly disseminate among vulnerable patients [223]. During the COVID-19 pandemic, shortages in personnel, personal protective equipment (PPE—e.g., gloves, gowns, and masks), and medical supplies led to deviations from standard IPC strategies, resulting in outbreaks of carbapenem-resistant *A. baumannii* [224]. Coordinated approaches, including IPC and ASPs, could prevent an estimated 619,000 HAIs over five years [225]. Moreover, the lack of rapid diagnostic tools in many healthcare settings means that antibiotics are often prescribed empirically, further contributing to the problem [226].

Antibiotic use in agriculture also plays a significant role in AMR emergence and dissemination [227]. In many countries, particularly the United States, Brazil, and China, antibiotics are widely used in livestock for the treatment and prevention of infections and to promote growth in food animals [25]. Antibiotics at sub-therapeutic doses often promote resistant bacteria’s emergence and selection, as well as their transmission to humans through the food chain, direct contact with animals, or environmental contamination [228].

In aquaculture, antibiotics are used to prevent and treat diseases [229], accumulating antibiotic residues in cultured aquatic products and developing bacterial resistance [230]. In crop production, especially in LMICs, antibiotic use is being recommended more frequently and for an increasing variety of crops [231]. The widespread use of antibiotics, coupled with the application of animal waste to agricultural land, contributes to antibiotic-related environmental contamination [232]. The environmental concentrations are generally below the therapeutic doses, but their long-term impacts on ecosystems, animals, and human health remain uncertain, requiring further research and stricter regulations [230,232].

Globalization and international travel have facilitated rapid AMR dissemination across borders [233]. Recent studies demonstrate that nearly 30% of international travelers acquire AMR bacteria during their trips to regions where resistant strains are prevalent and bring them back to their home countries, contributing to global AMR dissemination [234,235]. This is particularly concerning in pathogens such as *Neisseria gonorrhoeae*, where resistant strains have been globally disseminated and complicated treatment protocols [236]. Healthcare facilities should consider patients’ travel histories to implement appropriate treatment protocols and prevent further dissemination [233]. International surveillance and policy efforts are crucial to monitor and respond to this emerging global health crisis [233,234].

Medical tourism (i.e., individuals that travel to other countries for medical treatment) also contributes to AMR dissemination. Patients acquire resistant infections in healthcare settings abroad, particularly in countries with high AMR rates, and carry them back to their home countries [237]. It has been demonstrated that high-income countries (HICs) are more likely to receive AMR from LMICs, with Asia being a common origin for resistant bacteria—mainly enteric bacteria resistant to β-lactams and quinolones [238].

The environment acts as a reservoir for MDROs and ARGs due to the discharge of untreated or inadequately treated waste from hospitals, pharmaceutical factories, and agricultural operations into the environment [29]. These environmental MDROs can then enter human and animal populations, with anthropogenic activities contributing to their dissemination [239]. In high-income countries, human-to-human transmission is the primary route of AMR acquisition, while, in LMICs, inter-reservoir transmission is more common, mainly due to poor sanitation, a lack of clean water, and inadequate farming practices [239].

### 4.4. Comprehensive Strategies for Combating Antimicrobial Resistance

One of the most significant strategies to limit AMR emergence and dissemination is the implementation of ASPs in healthcare settings to promote rational antibiotic use [240]. These programs optimize clinical outcomes and healthcare costs and minimize AMR’s emergence and incidence by implementing several strategies (e.g., prospective audits with feedback, formulary restrictions, educational interventions, and rapid diagnostic tools) [241].

Strengthening the IPC strategies in hospitals and other healthcare facilities limits resistant organisms’ dissemination. Improving hand hygiene (HH), active screening for high-risk patients, isolating patients with resistant infections, and enhancing the use of PPEs are key components of this strategy [242]. Additionally, facility decontamination plays a crucial role in this multi-approach strategy. Decontaminating surfaces and equipment in healthcare settings can significantly reduce the environmental reservoirs of resistant pathogens, such as *C. difficile* spores, thereby lowering the risk of transmission and contributing to the overall effectiveness of antimicrobial resistance (AMR) control efforts. This measure complements other IPC strategies, ensuring a more comprehensive approach to combating AMR [243].

By monitoring AMR trends, global surveillance networks (e.g., GLASS and EARS-Net) guide public health interventions, control antibiotic use, and help to control AMR dissemination [244] by collecting antibiotic susceptibility data from clinical laboratories, assisting in developing diagnostics, vaccines, and novel antibiotics [244]. These programs face challenges in delivering consistent, high-quality data, especially in LMICs [245]. Alternative approaches are being explored to surpass these limitations. For instance, the use of sewage for AMR surveillance through metagenomic sequencing [245] provides affordable community-level surveillance in resource-poor settings, as revealed during the SARS-CoV-2 epidemic, when an integrated global AMR surveillance in wastewater environments was established [246,247]. As a result, several recommendations have been proposed for these programs. Reducing antibiotic use in agriculture, particularly their use as growth promoters, is vital in restraining AMR’s dissemination [133].

Another recommendation is to increase the public awareness of AMR’s dangers and educate healthcare professionals, farmers, and the public about responsible antibiotic use. Public health campaigns can change behavior by reducing unnecessary antibiotic use, a critical step to control AMR’s emergence and dissemination [133]. Investing in developing new antibiotics, alternative therapies, and rapid diagnostic tools allows us to stay ahead of this continuously evolving resistance. The development of vaccines and other preventive measures can reduce the need for antibiotics and help to control infection dissemination [133]. Finally, fostering global collaboration in research and innovation is essential to address AMR’s challenges [133].

## 5. Impact of Antimicrobial Resistance

AMR is one of the most pressing global health challenges of the 21st century, with profound consequences for human health, the effectiveness of medical treatments, and the economic stability of healthcare systems and societies worldwide [136]. According to an estimation, by 2050, AMR will be responsible for 10 million deaths annually, associated with a global economic burden of USD 100 trillion [240]. The ability of microorganisms to resist the effects of drugs intended to eliminate them leads to increased morbidity, mortality, and healthcare costs, posing significant challenges for public health [133].

### 5.1. Consequences of Antimicrobial Resistance for Human Health

The increase in AMR has severe implications for human health, primarily through increased morbidity and mortality [248]. Infections that were once easily treated with standard antibiotics have become more difficult to manage, leading to prolonged illnesses and higher risks of complications [249]. As a result, common infections (e.g., pneumonia, urinary tract infections, and bloodstream infections) are becoming increasingly resistant to first-line treatments, contributing to treatment failures and relapsing infections, further exacerbating health outcomes [250]. In France, an estimated 158,000 infections due to MDROs occurred in 2012, resulting in approximately 12,500 deaths [251]. Moreover, MDRO-caused HAIs are associated with higher mortality risks and readmission rates when compared to susceptible strains [252], as confirmed by a meta-analysis of studies in developing countries, particularly for the “ESKAPE” group pathogens [253]. The mortality rate for systemic infections with ESBL and MRSA is almost twice that of infections caused by susceptible pathogens [254]. As a result, AMR infections lead to increased healthcare costs, emphasizing the need for effective surveillance and prevention strategies to combat this growing global challenge [253].

Finally, AMR further complicates the management of chronic diseases and medical procedures. Patients undergoing chemotherapy, organ transplants, or major surgeries are at a higher risk of acquiring resistant infections due to their weakened immune systems [255]. In surgical settings, antibiotic prophylaxis prevents surgical site infections (SSIs), but increasing AMR raises the risk of SSIs complicated by resistant bacteria, leading to poorer outcomes [256].

### 5.2. Challenges in Treating Resistant Infections

Treating MDRO-caused infections is clinically challenging. The limited availability of effective antibiotics forces healthcare professionals to resort to less commonly used or more toxic alternatives, which may not be as effective and can lead to adverse side effects. In some cases, no effective treatment options may be available, leading to treatment failure and increased mortality [257].

One of the major challenges in treating resistant infections is the need for timely and accurate diagnosis [226]. Identifying the causative pathogen and its resistance profile is useful in selecting the appropriate treatment. The current diagnostic methods, primarily culture-based, are time-consuming and often lead to inappropriate antibiotic use [258]. Emerging technologies offer promising solutions (e.g., sample-in-answer-out PCR tests, rapid susceptibility tests, and sequencing-based approaches) and can guide targeted therapy, improve patient outcomes, and reduce AMR emergence [258,259]. Nevertheless, their implementation faces challenges, such as limited accessibility, high costs, and a lack of trained personnel [226]. Diagnostic stewardship, involving proper test selection and result interpretation, is essential for the optimal use of these tools [226]. Multidisciplinary collaboration and an increased awareness of the impact of good microbiology diagnostics are necessary to address these challenges and improve antibiotic use [226].

The emergence of MDR and extensively drug-resistant (XDR) organisms exacerbates the challenge of treatment. MDR pathogens, such as CRE, *A. baumannii*, and *P. aeruginosa*, are considered the highest-priority pathogens due to their ability to form biofilms and acquire multiple resistance genes [260]. The rise of XDR or “superbugs” (i.e., strains with resistance to most or all available antibiotics, resulting in extremely or totally drug-resistant phenotypes), such as MRSA, has led to an estimated 10,000 annual cases of nosocomial MRSA bloodstream infections in the United States [261,262,263].

The potential for treatment failure is particularly concerning in vulnerable populations (e.g., elderly or immunocompromised patients and those with underlying health conditions) [264], due to the body’s compromised ability to fight infections and the presence of resistant pathogens, which further diminishes the chances of recovery [265], impacting patients and placing a significant burden on healthcare systems. *C. difficile* exacerbates this issue, particularly among elderly and immunocompromised patients, leading to high morbidity and mortality and substantial economic burdens on healthcare systems worldwide. In the United States alone, CDI affects nearly 500,000 patients annually and results in around 30,000 deaths [265].

### 5.3. Economic Impact on Healthcare Systems and Society

The costs for healthcare systems are primarily attributed to longer hospital stays, intensive care expenditures, and more costly treatments for patients with resistant infections [266,267,268]. In Ireland, the AMR-associated excess length-of-stay costs were estimated at EUR 12 million in 2019 [269]. In France, the AMR costs exceed EUR 109 million, potentially reaching EUR 287 million if all cases were identified [270]. In the United States, the AMR infection treatment cost has doubled since 2002, reaching USD 2.2 billion annually (around USD 1400/patient) [270]. AMR infections increase mortality, with odds ratios of 1.844 (95% CI: 1.187–2.865) and higher readmission rates [271]. In France, the mean excess length of stay has been estimated at 1.6 days and 7.4 days (95% CI: 3.4–11.4) in a meta-analysis [269].

Beyond the direct healthcare costs, AMR presents other economic implications for society, mainly associated with productivity losses due to prolonged illness or premature death [272]. In Thailand, the estimated economic burden of AMR is USD 0.5 billion, and it is USD 2.9 billion in the US for five key pathogens [273]. LMICs bear a disproportionate burden, with AMR potentially reducing the global gross domestic product (GDP) by 1.1–3.8% annually by 2050.

The impact of AMR on the pharmaceutical industry is also noteworthy. The high cost of new antibiotic development, associated with the relatively short period of efficacy before resistance develops, has led to a decline in investment in antibiotic R&D, resulting in a dwindling pipeline of new antimicrobial agents and further exacerbating the AMR problem [274]. The high costs of clinical trials and the complexity of regulatory requirements further discourage companies from pursuing antibiotic development [161]. Governments and health agencies have attempted to incentivize reinvestment in antibiotic development, but the funding remains insufficient [275]. To address this crisis, there is a need for a broader scientific agenda, the better integration of industry and academia, streamlined regulatory paths, and new commercial models for antibacterial agents [275].

### 5.4. Global Implications and the Need for Coordinated Action

Addressing AMR requires collaboration among various stakeholders (e.g., healthcare professionals, policymakers, and the public), implementing strategies such as antimicrobial stewardship programs, controlling over-the-counter (OTC) sales, raising public awareness, establishing national guidelines for prudent antimicrobial use, sanitation improvements, and agricultural practices [13,276,277]. The fight against AMR requires collective responsibility and coordinated efforts at the local, national, regional, and international levels to ensure the sustained effectiveness of antimicrobials [278].

According to the WHO’s Global Action Plan (GAP), national action plans (NAPs) focusing on improving ASPs, enhancing IPC strategies, and increasing access to diagnostic tools and effective treatments are essential to combat AMR [279]. Many countries have developed NAPs that are fully aligned with the GAP’s objectives. Nonetheless, their implementation remains inconsistent, particularly in LMICs, due to limited resources, policy fragmentation, and “isomorphic mimicry”, where countries adopt policies without effective implementation [280]. To strengthen NAP implementation, it has been recommended that governance quality be improved, stakeholders be engaged, and actions across the human, animal, and environmental health sectors be coordinated [281]. A governance framework for NAPs has been proposed, focusing on policy design, implementation tools, and monitoring and evaluation [281]. International cooperation and legally binding responsibilities enhance global AMR governance and minimize policy fragmentation [280].

### 5.5. The Social and Ethical Dimensions of AMR

AMR extends beyond the clinical and economic impacts, creating important social and ethical issues. Social issues include balancing individual liberty with public health protection, addressing global distributive justice issues, and considering intergenerational responsibilities [282]. Recent studies have revealed a significant gap in understanding AMR’s social and structural drivers, with a limited focus on equity, gender, and social determinants of health [283]. These factors influence exposure, vulnerability, and the consequences of AMR, while also affecting the effectiveness of interventions [284]. A comprehensive approach is needed to ensure equitable access to antibiotics, improve living conditions, and challenge socioeconomic inequalities [285]. International cooperation is essential, as individual countries cannot combat AMR alone [286].

Ethical tensions arise between ensuring access to antibiotics in low-resource settings, curbing excessive use, and balancing personal interests against broader societal needs to combat AMR [287]. These issues (e.g., equity concerns, environmental impacts, and the role of pharmaceutical industries in drug development and promotion [288]) underscore the importance of a holistic, globally coordinated approach to combat AMR, involving multiple stakeholders and prioritizing equitable access to effective antimicrobials. Ethical issues also include antimicrobials’ use in agriculture and animal husbandry, which contributes to the emergence of resistant bacteria that can be transmitted to humans through the food chain. Balancing the need for food security and preserving the effectiveness of antibiotics for human health presents a significant ethical challenge [289].

## 6. Antimicrobial Stewardship and Infection Prevention Control

ASPs and IPC strategies are two key components of the global effort to combat AMR. While ASPs promote rational antibiotic use and preserve treatment effectiveness [290], improving patient outcomes and reducing healthcare costs [291], IPC strategies protect patients, healthcare professionals, and the broader community from infections and contribute to better patient outcomes, reduce healthcare costs, combat AMR, and ensure healthcare systems’ overall safety and trustworthiness [292].

### 6.1. Strategies to Promote Responsible Antimicrobial Use in Healthcare Settings

ASPs involve a systematic effort to optimize the use of antibiotics, ensuring that they are only used when necessary, in the correct dosage, and for the appropriate duration. Effective stewardship programs are essential in reducing the emergence of MDROs and improving patient outcomes. ASPs’ success relies on the intervention of multidisciplinary teams composed of infectious disease physicians and clinical pharmacists [241]. Several key strategies are employed in healthcare settings to promote responsible antimicrobial use.

One of the primary approaches is the implementation of guidelines with clear protocols for antimicrobials’ prescription. Often based on evidence-based practices, these guidelines are tailored to specific healthcare environments, such as hospitals, outpatient clinics, or long-term care facilities. By adhering to these guidelines, healthcare professionals can reduce the unnecessary use of antibiotics, which is a major driver of resistance [293].

Another critical aspect of ASPs is the role of antimicrobial stewardship teams (ASTs), including infectious disease specialists, pharmacists, microbiologists, and infection control experts [244], who are responsible for reviewing antibiotic prescriptions, providing feedback to prescribers, making recommendations for alternative treatments when appropriate, and educating healthcare staff about the risks of AMR and the importance of responsible antibiotic use [294]. Successful ASP implementation requires leadership, administrative support, and adaptation to the available resources in each healthcare facility [294].

Diagnostic stewardship is another essential component of responsible antimicrobial use [226]. By promoting the use of diagnostic tests and other technologies to quickly and accurately identify the causative pathogen, healthcare providers can tailor antibiotic therapies to the specific infection, reducing the use of broad-spectrum antibiotics and minimizing the risk of AMR development [295]. Molecular diagnostic tests (e.g., GeneXpert and GenoType MTBDRplus) identify the presence of specific ARGs (e.g., rifampicin and isoniazid), allowing the targeted treatment of infections, such as tuberculosis, within hours instead of days or weeks, as required for traditional culture methods [296].

Education and training programs for healthcare providers promote responsible antibiotic use and AMR management. These programs aim to improve prescribing practices and encourage the use of non-antibiotic alternatives when appropriate [297]. Moreover, patient education also helps to reduce the demand for antibiotics for viral infections, such as colds and the flu, where antibiotics are ineffective [298].

### 6.2. Importance of Infection Prevention and Control Strategies

**Figure 4:**
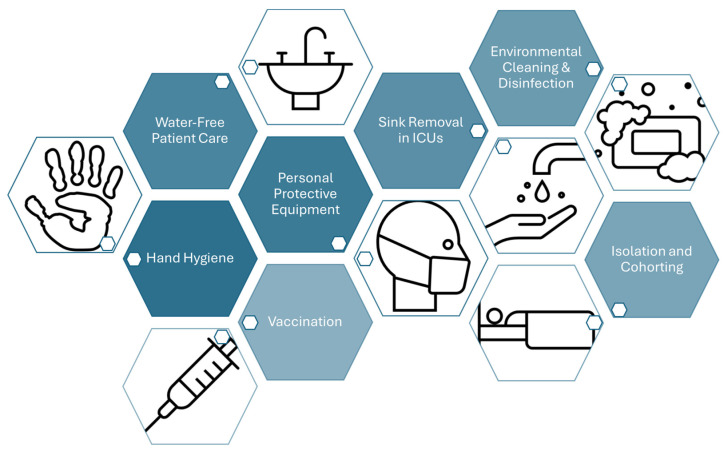
Effective IPC strategies to prevent MDRO emergence and dissemination.

IPC strategies not only reduce HAIs but also combat AMR. By preventing infections from occurring in the first place, the need for antibiotics is reduced, thereby limiting the opportunities for resistance to emerge and dissemination (Figure 4).

Compliance with HH, one of the most effective IPC strategies and considered as the cornerstone of infection control, remains suboptimal, especially among doctors [299]. Healthcare providers, patients, and visitors are encouraged to practice proper HH, using alcohol or chlorhexidine gluconate-based hand sanitizers or soap and water to prevent the transmission of pathogens [300]. Regular HH compliance audits and feedback are necessary to ensure adherence to this critical practice [301].

Another key IPC strategy is the use of PPE, particularly in situations where there is a high risk of infection transmission. Nevertheless, the use of PPE is often limited by several factors, such as discomfort, supply shortages, inadequate infrastructure, and a lack of training. To improve PPE adherence and effectiveness, healthcare facilities should develop context-driven implementation strategies, address healthcare personnel wellbeing, reorganize work processes, and adopt structural changes [302].

Water-free patient care aims to minimize or even eliminate water use in healthcare. In personal hygiene, pre-moistened disposable wipes replace traditional bed baths, ensuring patients’ cleanliness and comfort. Water-free dressings and sprays replace conventional saline solutions in wound management, reducing infection risks. Alcohol-based hand sanitizers replace soap and water, improving HH and infection control. The benefits of this innovative approach include environmental sustainability through reduced water usage, coupled with improved infection control and enhanced patient comfort [303].

Sink removal, mainly in intensive care units (ICUs), has been proven efficient in healthcare settings. Sinks constitute reservoirs for harmful bacteria, including MDROs, which are easily disseminated to the surrounding environment. By eliminating sinks, the contamination risk decreases, reducing HAIs. Moreover, in the absence of sinks, healthcare professionals rely on alternative HH practices that have been proven to be more effective, leading to better HH compliance among staff and reducing the likelihood of infection transmission [304].

Environmental cleaning, disinfection, and decontamination are critical components of IPC. The regular cleaning and disinfection of high-contact surfaces, medical equipment, and patient care areas are essential to eliminate pathogens and reduce the risk of infection [305]. Traditional manual cleaning methods are often insufficient, highlighting the need for advanced solutions such as gas generation systems and fogging equipment capable of producing a homogeneous cloud of decontaminants, such as peroxide-based disinfectants. These methods can reach difficult-to-access areas where pathogens may proliferate, contributing to environmental reservoirs in healthcare facilities and other settings. Additional approaches, such as electrolyzed water and “no-touch” automated systems such as UV-C light devices, are valuable in a multifaceted strategy to control AMR [306]. These procedures are particularly important since several pathogens of concern for IPC can persist on inanimate surfaces for periods of time that range from weeks to almost two years (*K. pneumoniae*: 600 days; *S. aureus*: 318 days; *C. difficile*: 140 days; *Acinetobacter* spp. 90 days; *E. coli*: 56 days; Coronavirus: 20 days; *C. auris*: 14 days) [307].

The isolation and cohorting of patients with known or suspected resistant infections are additional strategies to prevent MDRO dissemination [308]. Nevertheless, isolation may negatively affect patient outcomes, including longer hospital stays, higher costs, and increased readmission rates. Moreover, isolation implementation may be challenging in LMICs due to resource constraints [309].

Vaccines prevent targeted pathogen infections, protect against resistant subtypes, and decrease bystander selection [310]. The impact of vaccination on AMR has been demonstrated for vaccines against *Streptococcus pneumoniae*, *Haemophilus influenzae* type b, and influenza [303]. Despite the GAP recommendation on vaccination, its adoption in NAPs is not universal, particularly in LMICs [311], due to challenges such as the National Immunization Technical Advisory Group (NITAG) capacity, access to evidence, and resources for systematic vaccine policy decision making [312]. The development of vaccines against resistant pathogens faces challenges due to pathogens’ complexity and technical difficulties [313]. Mathematical models suggest that the impact of vaccination on antibiotic resistance depends on the relative strength and directionality of the competition between drug-resistant and drug-sensitive strains, emphasizing the need for tailored policies [314].

### 6.3. Global Surveillance Systems for Antimicrobial Resistance

Surveillance is essential to the global response to AMR, guiding interventions, informing policies, and tracking stewardship and IPC strategies’ effectiveness [315,316]. Different surveillance approaches exist, from traditional clinical sample analysis to sewage-based surveillance using metagenomic sequencing [245]. Surveillance systems operate at different levels, from local to international, providing data on AMR prevalence and distribution. Effective surveillance requires timely feedback to stakeholders and correlation with demographic and clinical data to understand the epidemiology and formulate interventions [317].

National surveillance programs, such as the United States Centers for Disease Control and Prevention’s (CDC) National Healthcare Safety Network (NHSN) and the European Antimicrobial Resistance Surveillance Network (EARS-Net), collect data from healthcare facilities across their respective regions to monitor resistance trends, identify emerging threats, and provide benchmarks to compare resistance rates between different locations [315]. The Study for Monitoring Antimicrobial Resistance Trends (SMART) is a long-running (i.e., 20 years), industry-sponsored program that has collected data from over 60 countries, providing valuable information for treatment guidelines and research [317]. In the Netherlands, a successful national AMR surveillance system based on routine data from medical microbiological laboratories has been supporting policymaking and guideline development since 2008 [318].

At the international level, GLASS coordinates efforts to monitor AMR by collecting and sharing data from participating countries, providing a global picture of resistance patterns and trends [319]. Despite the challenging implementation of these programs in LMICs, guidelines have been developed to facilitate their participation [320]. In Thailand, data collection is challenging and specimen contamination often occurs, highlighting the need for additional training [321]. A recent analysis of GLASS data demonstrated significant correlations between antimicrobial consumption and resistance rates for certain pathogens and antibiotics across participating countries [322]. This information can be used to identify global AMR hotspots, assess intervention impacts, and guide international efforts to combat AMR [319,322].

Surveillance data help in developing APSs across healthcare settings. By identifying the most prevalent resistant pathogens and their antibiotic resistance profiles, healthcare professionals can make informed decisions on the most appropriate treatment options, avoiding inappropriate antibiotic use and not only preserving the existing antibiotics’ effectiveness but also reducing AMR dissemination [323].

### 6.4. Novel Approaches to Combat Antimicrobial Resistance

There is an urgent need for novel approaches to combat AMR and preserve antimicrobial therapies’ effectiveness. Several promising strategies are currently being explored, including the development of new antibiotics, alternative therapies, and innovative technologies (Table 2).

One of the most critical areas of research is the development of new antibiotics with novel mechanisms of action. Traditional antibiotics often target essential bacterial processes (e.g., cell wall synthesis and protein production). For instance, teixobactin, a novel antibiotic isolated from uncultured bacteria with potent activity against Gram-positive pathogens, destroys bacteria through a two-pronged attack on the cell envelope by targeting cell wall precursors (Lipids II and III) and disrupting the membrane [324].

Bacteriophages are emerging as an alternative to traditional antibiotics due to their high specificity, ecological safety, and potential applications beyond clinical settings [325,326]. Nevertheless, this alternative presents some challenges, mainly associated with large-scale production, formulation, stability, and bacterial resistance to phages [326]. Another phage-related innovative approach involves exploiting phage–bacteria interactions to discover new antibacterial targets and develop small molecules that mimic inhibitory phage proteins [327].

Antimicrobial peptides (AMPs) constitute another promising candidate in combating AMR due to their broad-spectrum activity and lower propensity for resistance development [328]. These naturally occurring molecules, found in most multicellular organisms, exhibit potent antibacterial, antiviral, and antifungal properties and are capable of disrupting cell membranes [329]. Despite their ancient origin, AMPs represent a promising therapeutic strategy against antimicrobial resistance, with several peptide-based drugs already approved by the U.S. Food and Drug Administration (FDA) [330]. However, further research is required to understand AMPs’ action mechanisms and optimize their clinical applications.

Alternative therapies, such as immunotherapies and probiotics, are also being explored as potential strategies to combat AMR. Monoclonal antibodies are promising due to their high specificity and bacteria’s inability to develop resistance [331]. These antibodies neutralize bacterial toxins and virulence factors [332]. Probiotics and fecal transplant therapy are being studied to enhance the human microbiota and prevent infections [333]. Pathogen-oriented therapy (POT) is emerging as a targeted approach to circumvent AMR, utilizing antibiotic–antibiotic conjugates [334], nanotechnologies, and CRISPR-Cas systems [335]. Combination therapies and the repurposing of existing drugs are also being explored [336].

Nanoparticle-based technologies have been shown to be promising to overcome resistance in planktonic and biofilm phenotypes, by combining novel nanomaterials with classic antimicrobial therapies [337]. Nanoparticles enhance antibiotic activity, preserve existing antibiotics’ efficacy, and provide novel antibacterial action modes [338].

AI applications include predictive modeling, rational antibiotic use, and the development of antimicrobial peptides and antibiotic combinations [339]. Machine learning techniques are accelerating new antibiotic discovery, optimizing treatment regimens, and predicting resistance patterns [340]. These AI-driven approaches focus on predicting antimicrobial activity and drug-likeness traits and enabling de novo molecular design. The integration of these technologies offers promising avenues to address the current AMR crisis.

Finally, there is a growing emphasis on the One Health approach to combat AMR. International organizations, such as the WHO, ECDC, and World Organisation for Animal Health (OIE), play a significant role in promoting the One Health approach to AMR, preserving the effectiveness of existing antimicrobials, and limiting infection dissemination across the human, animal, and environmental domains [341,342,343,344].

## 7. The One Health Approach to Antimicrobial Resistance

The One Health approach has emerged as a comprehensive strategy to combat AMR by recognizing the interconnectedness of human, animal, and environmental health. This approach emphasizes the need for collaborative efforts across various sectors to address the complex and multifaceted challenges posed by AMR [341,342,343,344].

### 7.1. Recognition of the Interconnectedness of Human, Animal, and Environmental Health

The interconnectedness between the health of humans, animals, and ecosystems, supported by the One Health approach, is particularly evident in the context of AMR, where the use of antimicrobials in one domain profoundly affects others. The overuse or misuse of antibiotics in agriculture, human medicine, and veterinary care contributes to MDROs’ emergence and dissemination among these different system parts [341,342,343,344].

Many countries have implemented NAPs based on the One Health approach, following guidelines from international organizations (e.g., WHO, OIE, and Food and Agriculture Organization of the United Nations—FAO), aiming to mitigate AMR dissemination, raise awareness about antibiotic use, and promote antimicrobial stewardship [345].

#### 7.1.1. The Human Health Sector

In hospitals and outpatient clinics, antibiotics are frequently prescribed for conditions where they are not needed, such as viral infections [220], leading to the selection of resistant strains of bacteria, which can disseminate within the community and healthcare facilities, causing difficult-to-treat infections [346]. Patient expectations and misconceptions contribute to this issue, as 53% of patients incorrectly believe that antibiotics are effective against viral infections [347]. Clinicians often prescribe antibiotics due to perceived patient demands, concerns about patient satisfaction, and a fear of negative repercussions after denying antibiotics [348]. Effective clinician–patient communication is essential to provide clear explanations, use specific diagnoses, and offer alternative treatments to reduce unnecessary antibiotic prescription while maintaining patient satisfaction [349].

#### 7.1.2. The Animal Health Sector

The widespread use of antibiotics in animal husbandry, for therapeutic purposes or as growth promoters, creates a reservoir of resistant bacteria in animals that can be transmitted to humans through the food chain or direct contact, such as *Salmonella*, *Campylobacter*, *E. coli*, *S. aureus*, and *C. difficile* [350]. Some countries have banned the use of antibiotics as growth promoters and are focusing on developing alternatives (e.g., vaccines, probiotics, and bacteriophages), including new biosafety–biosecurity and management approaches, to reduce AMR dissemination among animals in farms [351,352].

#### 7.1.3. The Environmental Health Sector

Antibiotics and antibiotic-resistant bacteria may enter the environment through various pathways (e.g., agricultural runoff, wastewater discharge, and improper disposal of pharmaceuticals) [239], and, once in the environment, they can persist and disseminate, affecting wildlife and entering the human food chain. The presence of antibiotics in water bodies, soil, and sediments promotes the selection of resistant organisms in these ecosystems, creating reservoirs of resistance that can be transmitted back to humans and animals [29]. The agricultural ecosystem facilitates AMR emergence through the indiscriminate use of antibiotics in agricultural and veterinary practices [353]. While HICs have more controlled discharges, LMICs face greater challenges due to poor sanitation and deficient farming practices [239]. Addressing environmental AMR requires improved waste treatment, reduced antibiotic use, and better pharmaceutical waste control [239].

### 7.2. Importance of Collaborative Efforts between Healthcare, Veterinary, and Environmental Sectors

The One Health approach emphasizes the importance of collaboration and coordination among various sectors to effectively combat AMR. By working together, stakeholders from healthcare, veterinary medicine, agriculture, and environmental management can develop and implement strategies that address the causes of resistance and mitigate its impact on public health [341].

#### 7.2.1. Assessing One Health Integration in Antimicrobial Resistance Surveillance Systems

One of the key components of the One Health approach is the establishment of integrated surveillance systems that monitor the use of antimicrobials and resistance dissemination across the environmental, animal, and human domains. Unfortunately, many of these programs are often fragmented and lack full integration [354]. A review of electronic information systems (EIS) for AMR surveillance found no systems that fully integrated data across all three sectors, highlighting a significant gap in global efforts to address AMR [355]. A conceptual framework has been developed to evaluate the integration of the One Health approach in AMR surveillance systems, focusing on five key components: the capacity to integrate a One Health approach, produce One Health information, generate actionable knowledge, influence decision making, and impact outcomes [356].

#### 7.2.2. Collaborative Policy Approaches to Combat Antimicrobial Resistance

Effective policies to combat AMR require collaboration between environmental, veterinary, and healthcare policymakers to develop regulations promoting several measures [33], such as ASPs in veterinary and human medicine [357], environmental regulations limiting antibiotic release in ecosystems [33], and the harmonization of regulatory frameworks across countries [13]. Challenges include balancing individual ministry priorities with cross-cutting issues and building mutual trust [357]. Immediate actions required include establishing ASPs, controlling OTC sales, and promoting public awareness [13]. A global fund and international collaboration integrating social, economic, and behavioral factors are recommended to address AMR effectively [13].

#### 7.2.3. Joint Research and Innovation

The One Health interdisciplinary approach encourages collaborative research efforts to develop innovative solutions to combat AMR [358,359]. The exploration of bacteriophages as potential alternatives to antibiotics in human and veterinary medicine has been shown to be a promising alternative, due to their highly specific and potent antimicrobial activity against resistant species [326], although challenges remain in their large-scale production and implementation [326].

#### 7.2.4. Education and Awareness

Various educational activities, including games, roleplay, and e-learning, can effectively engage diverse audiences in understanding this phenomenon [360]. While healthcare professionals across the animal and human sectors often demonstrate high AMR awareness, this knowledge does not necessarily translate into reduced antibiotic prescribing [361].

#### 7.2.5. Strengthening Infection Prevention and Control

IPC strategies are essential in reducing resistant pathogens’ dissemination across environmental, animal, and human interfaces. In healthcare settings, IPC practices (e.g., HH, PPE use, environmental cleaning, and routine decontamination) prevent the transmission of resistant bacteria [341]. In veterinary medicine, biosafety and biosecurity measures, vaccination, and proper animal husbandry practices reduce the incidence of infections in livestock, thereby decreasing the need for antibiotics [362]. Environmental management practices (e.g., proper waste disposal and the treatment of agricultural runoff) limit resistant organisms’ dissemination in the environment [363].

#### 7.2.6. Global Cooperation and Capacity Building

LMICs often face significant challenges in addressing AMR, such as limited healthcare access, weak regulatory frameworks, and inadequate infrastructure for surveillance and laboratory testing [364]. International cooperation among the WHO, OIE, and FAO builds the capacity in these countries by providing technical assistance, funding, and access to expertise [365]. Global cooperation also involves sharing best practices, data, and resources and coordinating efforts to address AMR in transboundary contexts, such as controlling resistant pathogens’ dissemination through international travel and trade [365].

## 8. Policy and Regulatory Initiatives

In response to the growing threat of AMR, international and national policymakers have developed a range of strategies and regulatory frameworks to combat AMR, promote responsible antimicrobial use, and preserve the effectiveness of existing treatments.

### 8.1. Overview of National and International Policies

The recognition of AMR as a transboundary issue that requires a global response has led to the formulation of several AMR-oriented international and national policies focusing on several key areas, such as surveillance, ASPs, R&D, and public awareness [126].

#### 8.1.1. International Policies

International organizations (e.g., WHO, OIE, FAO) have taken the lead in coordinating global efforts to combat AMR [366]. In 2015, the WHO launched the GAP on AMR, which encouraged more than 100 countries to develop their own NAPs to address this issue [280]. The GAP outlines five strategic objectives that should be implemented in NAPs: improving the awareness and understanding of AMR, strengthening surveillance and research, reducing the incidence of infection, optimizing the use of antimicrobial agents, and fostering sustainable investment in new medicines and diagnostic tools, vaccines, and other interventions [280]. The WHO also oversees the GLASS, which collects and analyzes AMR data from member states, aiming to strengthen national surveillance systems and facilitate data sharing among countries [319]. Despite the guidelines developed to ensure data validity and comparability, the implementation of this system in LMICs faces several challenges, as previously described [320,321].

The OIE has developed standards for the responsible use of antimicrobials in veterinary medicine, which are integrated into the national regulations of its member countries [367]. The OIE has also created the List of Antimicrobial Agents of Veterinary Importance, which collects data on antimicrobial use and supports member countries in implementing standards through various programs [367].

The FAO has focused on reducing antibiotic use in agrifood systems through programs to strengthen governance, increase awareness, enhance AMR surveillance, and implement best practices in food production [368]. Developing countries face challenges in controlling AMR in the food chain, necessitating comprehensive measures from farm to fork [253]. Environmental factors (e.g., soil and water contamination) also contribute to ARG dissemination in food production systems [369].

#### 8.1.2. National Policies

Many countries have developed NAPs in line with the WHO’s GAP, and these typically include measures to enhance surveillance, promote antimicrobial stewardship, support R&D, and raise the public awareness of AMR [279,280]. While strong vertical alignment exists between the GAP and NAPs, particularly in LMICs, horizontal alignment within regions is weaker [280]. NAPs generally align well with the GAP objectives but less with recommended actions [279]. Therefore, gaps exist in monitoring and evaluation, financing plans, and targets for antimicrobial use reduction [370]. The United Kingdom’s “UK Five Year Antimicrobial Resistance Strategy” emphasizes the importance of improving IPC strategies, optimizing the use of antimicrobials in animal and human medicine, and investing in new treatments and diagnostics [371]. Similarly, the United States’ “National Action Plan for Combating Antibiotic-Resistant Bacteria” aimed to establish ASPs in all acute care hospitals by 2020 [372]. In China, an NAP significantly reduced the AMR rates and culture positivity in a teaching hospital [373]. In Tanzania, a year after launching their NAP, the implementation of AMR surveillance and ASPs was found to be insufficient and inconsistent across healthcare facilities, highlighting areas for improvement in addressing AMR [374].

In many LMICs, NAPs often focus on strengthening the healthcare infrastructure, improving access to point-of-care treatment [375], and ensuring the availability of quality medicines (i.e., avoiding substandard or falsified medicines). However, challenges such as limited resources, weak regulatory frameworks, and inadequate surveillance systems can hinder the effective implementation of these policies [376].

### 8.2. Regulatory Frameworks to Promote Responsible Antimicrobial Use

Regulatory frameworks ensure the responsible use of antimicrobials and preserve their effectiveness, and they typically encompass regulations on antibiotics’ sale and use, guidelines for healthcare professionals, and measures to control antimicrobial use in agriculture and veterinary medicine.

#### 8.2.1. Regulatory Frameworks and Antimicrobial Stewardship in Combating Antibiotic Misuse in Human Health

In the healthcare sector, regulatory frameworks often include guidelines for the prescription of antibiotics, OTC sales restrictions, and measures to prevent the misuse of antimicrobials. In countries such as India [377], Nepal [378], Thailand [379], Ghana [380], and Zambia [380], OTC sales remain as a common practice, with amoxicillin, azithromycin, and ciprofloxacin commonly sold for respiratory issues and diarrhea [381,382]. In contrast, in other countries, such as Portugal, antibiotics are prescription-only medicines, meaning that they can only be dispensed with a valid prescription from a licensed clinician, preventing antibiotic overuse and misuse, considered major drivers of AMR [383].

Regulatory bodies (e.g., the FDA and the European Medicines Agency—EMA) ensure the safety and efficacy of antimicrobial drugs by evaluating new agents, monitoring their use, and publishing guidelines for healthcare professionals on appropriate prescribing practices [234]. These agencies also oversee ASP development and implementation, which is designed to optimize the use of antibiotics in healthcare settings [384]. ASPs often include educational initiatives to inform healthcare professionals about the AMR risks and the importance of judicious antibiotic use. They may also involve establishing multidisciplinary teams (e.g., pharmacists, infectious disease specialists, and microbiologists) to guide antibiotic prescribing and monitor patient outcomes [294].

#### 8.2.2. Regulatory Frameworks and Antimicrobial Stewardship in Combating Antibiotic Misuse in Animal Health and Agriculture

Regulatory frameworks in the agriculture and veterinary sectors typically focus on reducing the use of antibiotics as growth promoters, limiting their use for prophylactic purposes, and promoting best practices in animal husbandry to prevent infections. In the European Union (EU), the use of antibiotics as growth promoters in livestock was banned in 2006, while countries such as Denmark and the Netherlands have successfully reduced veterinary antibiotic usage [385]. Alternative approaches to antibiotics in livestock (e.g., bacteriophages, engineered peptides, probiotics, and quorum-quenching molecules) have shown promising results [386]. Improved biosecurity measures, animal husbandry practices, and farm management have also reduced antibiotic use in many countries [387].

In 2014, the FDA curbed the use of medically important antibiotics as growth promoters in livestock [388] and implemented guidelines for the judicious use of antibiotics (e.g., banning growth promotion applications and requiring veterinary oversight for feed and water administration) [389]. The FDA’s approach also includes a qualitative risk assessment for new animal drugs to evaluate their potential for the development of resistance in bacteria harmful to humans [390]. However, challenges remain in surveillance and regulatory oversight; while the poultry industry has significantly reduced its antibiotic use, their usage in the pork and beef industries remains considerable [389].

#### 8.2.3. Regulatory Frameworks and Antimicrobial Stewardship in Combating Antibiotic Misuse in Environmental Health

Various drivers contribute to AMR dissemination, including antibiotic use in healthcare and agriculture and the release of antibiotic residues into soil, water, and air [391]. Pharmaceutical effluents, particularly in LMICs, pose significant risks to ecosystems and public health [392]. Critical knowledge gaps exist regarding the sources of antibiotics and ARGs in the environment, the role of anthropogenic inputs in resistance evolution, the health impacts of environmental exposure, and the efficacy of mitigation strategies [28]. Addressing AMR requires a collaborative One Health approach involving policymakers, regulators, manufacturers, and researchers to develop global standards for pharmaceutical effluent management, improve treatment methods, and implement comprehensive national action plans [391,392]. The EU’s Water Framework Directive (WFD) addresses the problem of AMR in aquatic environments by setting environmental quality standards for antibiotic concentrations [393]. To guide regulatory efforts, predicted no-effect concentrations for resistance selection have been proposed for common antibiotics, ranging from 8 ng/L to 64 μg/L [132]. These concentrations are often lower than ecotoxicological thresholds, highlighting the need for specific regulations addressing antibiotic resistance. The presence of antibiotics in aquatic ecosystems negatively impacts microbial flora and promotes ARG dissemination [394]. The implementation of the WFD to control antibiotic pollution could drive other legislation to consider AMR-associated risks.

### 8.3. Challenges and Opportunities for Implementation of Policy Interventions

While significant progress has been made in developing policies and regulatory frameworks, several challenges remain in their implementation. These challenges include limited resources, a lack of political will, a weak regulatory capacity, and the need for global coordination.

#### 8.3.1. Resource Limitations

One of the primary challenges in implementing AMR policies is the lack of resources and infrastructure, particularly in LMICs [379,395]. Many countries face significant barriers in establishing robust surveillance systems, implementing ASPs, and enforcing regulations. Limited access to healthcare, inadequate laboratory infrastructure, and insufficient funding for public health initiatives hinder the effective implementation of AMR control policies [364]. To address these challenges, countries must strengthen their health systems, improve their technical capacities, and enhance their governance for effective multi-sectoral action [379]. AMR surveillance systems with integrated models combining clinical, laboratory, and demographic data are more informative than laboratory surveillance alone [323].

#### 8.3.2. Political Will and Regulatory Capacity

The successful implementation of AMR policies depends on political will and the regulatory capacity. In some countries, there may be resistance in adopting strict regulations on antibiotic use, particularly in agriculture, due to concerns about the economic impact and industry opposition. Weak regulatory frameworks and a lack of enforcement capacity can further undermine efforts to control AMR [396].

Building political will requires advocacy and public awareness campaigns highlighting AMR’s long-term effects and economic costs [13]. Engaging stakeholders from various sectors, including healthcare, agriculture, and the pharmaceutical industry, can help to build a consensus on the need for action [33]. Strengthening regulatory agencies and ensuring adequate funding for enforcement activities ensures the successful implementation of AMR policies [397].

Public awareness campaigns about the long-term effects and economic costs of AMR are necessary [398]. However, challenges such as insufficient public awareness, limited community engagement, and inadequate human resources hinder this progress [397]. Opportunities for improvement include developing coordinated surveillance systems and capitalizing on existing structures and practices to manage the antimicrobial supply and prescription [33,397].

#### 8.3.3. Global Coordination and Harmonization

The disparities in regulatory frameworks, surveillance systems, and enforcement capacities can create challenges for global coordination. For instance, while some countries have banned the use of antibiotics as growth promoters in livestock, others continue to allow this practice, creating a potential source of resistant bacteria that can be disseminated across borders.

International organizations (e.g., WHO, OIE, and FAO) promote harmonization and facilitate information exchange [399]. However, a comprehensive “whole United Nations approach” to AMR is still lacking, with only 21 out of 78 assessed organizations having AMR-specific activities [366]. The FAO supports nations in improving ASPs through various programs, including developing a platform to collect data on AMR in animals and antimicrobial use in plants [368]. While there is strong vertical alignment between NAPs and the GAP, particularly among LMICs, the horizontal alignment within regions is weaker. The implementation of NAPs often lacks policy alignment, suggesting a need for legally binding global governance initiatives [280].

#### 8.3.4. Opportunities for Innovation and Collaboration

Despite the challenges, there are significant opportunities for innovation and collaboration in AMR management. Public–private partnerships, such as CARB-X, are accelerating the development of new antibiotics, vaccines, and diagnostics [400]. Advances in understanding bacterial physiology, clinical trial designs, and regulatory pathways enable the development of pathogen-targeted antibiotics responsible for patient outcome improvements and ASPs [401]. To address the declining antibacterial pipeline, a broader scientific agenda, better industry–academia integration, and a new commercial model are needed [154]. Horizon scanning methods have identified more than 3000 innovative AMR prevention, detection, and monitoring technologies, focusing on diagnostic innovations for the rapid and accurate detection of drug-resistant infections [402]. These technological advancements present opportunities to strengthen national AMR strategies and support appropriate antimicrobial prescribing.

## 9. Future Directions and Challenges

AMR has rapidly evolved from a manageable health concern into a threatening global crisis. The ability of pathogens to develop antimicrobial resistance undermines decades of progress in medicine, rendering common infections potentially lethal and complicating medical procedures. As the world struggles with this escalating threat, it is essential to explore the emerging challenges, identify research priorities, and consider the broader societal and ethical implications of the fight against AMR.

### 9.1. Emerging Threats in Antimicrobial Resistance

#### 9.1.1. Multidrug-Resistant Pathogens

One of the most alarming developments in the realm of AMR is the rise of MDROs (e.g., MRSA, CRE, MDR-TB, and multidrug-resistant *C. difficile*) capable of resisting multiple classes of antimicrobials, severely limiting the treatment options and leading to higher mortality rates [393]. MDROs’ dissemination in healthcare settings and the community highlights the urgent need for robust IPC strategies and the development of new treatment options.

#### 9.1.2. Novel Resistance Mechanisms

The emergence of novel antibiotic resistance mechanisms also poses significant challenges to public health. The *mcr-1* gene, conferring resistance to colistin, has rapidly disseminated worldwide, mainly by its capacity to be transferred by HGT [403,404]. ESBLs contribute to resistance against a wide range of β-lactam antibiotics, while metallo-β-lactamases (MBLs) are a major concern, particularly in carbapenem-resistant Gram-negative bacteria, such as *K. pneumoniae*, *A. baumannii*, *P. aeruginosa*, *E. coli*, and *Enterobacter* species [405]. The MCR-1 protein, a phosphoethanolamine transferase, has been extensively studied regarding the potential development of MCR-1 inhibitors for use in combination therapies [406].

### 9.2. Research Priorities for New Antimicrobial Agents and Alternative Strategies

#### 9.2.1. Development of Novel Antimicrobials

Given the declining efficacy of existing antibiotics, there is an urgent need to develop novel antimicrobial agents. Research priorities include identifying new drug targets and mechanisms of action [407]. The discovery of teixobactin, a novel antibiotic with a unique mechanism of action, exemplifies the potential for breakthroughs in this area [408]. However, economic challenges have delayed progress in antibiotic development, requiring innovative financial models to incentivize pharmaceutical companies [409]. Alternative explored strategies include combination therapy, the targeting of resistance mechanisms, and novel drug delivery systems [407]. According to the WHO’s reports, only 60 antibacterial drugs are currently under clinical development, with only six meeting this novel criterion [410]. Other promising approaches include nanotechnology, computational methods, and drug repurposing [411]. Non-traditional alternatives (e.g., antimicrobial peptides, bacteriophages, and prodrugs) are also being investigated [411]. Continued research, public investment, and regulatory reforms are essential to address the antibiotic development crisis [410].

#### 9.2.2. Alternative Treatment Strategies

There is growing interest in alternative treatment strategies that can enhance the effectiveness of existing drugs or provide entirely new approaches to combat AMR. These include phage therapy, AMPs, immunotherapy, and microbiome modulation.

Bacteriophage therapy has re-emerged as a promising alternative to antibiotics [412]. Phages are viruses that specifically target and kill bacteria, offering an environmentally friendly approach to controlling pathogens in agriculture and aquaculture [413]. Their unique mechanism of action and cost-effective production make them ideal in addressing AMR infections, especially in LMICs [412]. Phages have also demonstrated effectiveness against various foodborne and zoonotic pathogens (e.g., *Salmonella*, *E. coli*, and *Listeria*) [414]. Historically, phage therapy has been used since 1919, with successful treatments for dysentery and other bacterial infections [415]. Despite these challenges, ongoing research focuses on expanding clinical trials, standardizing the production and storage of phage cocktails, and promoting international collaboration to advance phage therapy as a solution to AMR [412].

AMPs, essential innate immune system components, exhibit broad-spectrum antimicrobial activity against several microorganisms [416], being promising therapeutic agents due to their low resistance induction and ability to modulate the immune response [417]. However, their clinical use is limited by their toxicity to mammalian cells, reduced activity under physiological conditions, and susceptibility to proteolytic degradation [418]. Research should focus on optimizing AMPs through modifications and multimerization to improve their antimicrobial activity, specificity, and biocompatibility [417]. Nanomedicine approaches are being explored to develop targeted delivery systems for AMPs, potentially increasing their efficacy and reducing their toxicity [419].

Recent research considers boosting host immune responses to combat infections, particularly in the context of respiratory diseases. Innate immunity, including antimicrobial peptides and ROS, defends against pathogens and regulates the microbiota [420]. Host-directed therapies aim to enhance the innate defenses, especially against ARB. Toll-like receptor (TLR) agonists have shown promise in triggering trained immunity, enhancing antimicrobials’ functions through metabolic and epigenetic modifications [421]. Modulating the host response has emerged as a potential strategy for severe lung infections to mitigate damage and prepare for future pandemics [422]. However, some bacteria can exploit the anti-inflammatory responses in the lungs to evade elimination. Targeting these bacterial-induced immunosuppressive mechanisms could improve the treatment options for chronic lung infections [423]. These approaches offer pathogen-agnostic strategies to enhance the host defenses against various infections.

The human microbiome plays a role in health and disease, while its modulation offers potential therapeutic benefits [424]. Strategies to manipulate the microbiome include probiotics, prebiotics, postbiotics, and fecal microbiota transplantation (FMT) [425]. These interventions are being investigated for various conditions, including infections and complications associated with allogeneic hematopoietic cell transplantation [426]. Probiotics, prebiotics, and postbiotics have shown promise in preventing and treating diseases, although their use is currently limited to dietary supplements [424]. The rise of AMR pathogens has led to increased interest in microbiota-based treatments as alternatives to traditional antibiotics [427]. However, challenges remain in defining precise microbiome targets, refining interventions, and standardizing the outcome measures across studies [425]. Further research is needed to fully understand the microbiome’s role in various diseases and optimize its modulation for therapeutic purposes.

### 9.3. Societal and Ethical Implications of Antimicrobial Resistance Interventions

#### 9.3.1. Access and Equity

Antimicrobial resistance disproportionately affects LMICs, exacerbating health inequalities [288]. Therefore, addressing AMR requires a holistic approach that considers its ethical components, such as equitable access to antibiotics and the role of social determinants of health [288]. Current approaches often focus on individual behavior change, but AMR is deeply embedded in societal values and culture, necessitating a shift toward collective responsibility [428]. The One Health approach is essential in AMR studies but often remains anthropocentric in practice [429]. Recently, a more-than-human ethical framework based on relationality and care ethics has been proposed to address these challenges [429]. Successful AMR mitigation will require collaboration at the individual, community, and national levels to ensure the continued effectiveness of antimicrobials [398].

#### 9.3.2. Stewardship and Overuse

Although ASPs aim to optimize antibiotic use, they fail to balance patient care with resistance prevention [165]. The drivers of broad-spectrum antibiotic overuse include individual, social, and structural factors, which vary across healthcare contexts [430]. Ethical considerations in antibiotic use and distribution encompass issues of equity, access, and environmental impacts [288]. Solutions to improve stewardship include increasing practitioner responsibility, enhancing caregivers’ roles as diagnosticians, improving patient communication, reducing economic influences on prescribing, and identifying antibiotic alternatives [431]. The WHO has developed a toolkit for the implementation of ASPs, particularly in LMICs, where the AMR burden is high and resources are limited [156].

#### 9.3.3. Environmental Impact

AMR requires a One Health approach, recognizing the interconnectedness of human, animal, and environmental health [344]. The environment, especially water resources, constitutes the primary reservoir and dissemination pathway for AMR [432]. Inadequate antibiotic use in the agriculture, veterinary, and medical sectors contributes to the spread of ARB and ARGs in soil, air, water, and sediments [13]. Agricultural soils are a major sink for antibiotics and AMR from livestock farming, with manure application augmenting soil resistomes [25]. The current global and national ASPs primarily focus on mitigating antibiotic use in the human and animal sectors, overlooking environmental drivers [432]. Addressing AMR requires the identification of high-risk environments; improvements in infection control, sanitation, and access to clean water; and the regulation of antimicrobial use in agriculture [13].

#### 9.3.4. Ethical Dilemmas in Research and Innovation

The development of new antimicrobial treatments, mainly phage therapy, presents significant ethical dilemmas. Despite showing promising results, phage therapy faces challenges related to regulatory frameworks and clinical trial designs [433]. Ethical concerns arise from the manipulation of viruses that may evolve unpredictably and the complexities of informed consent in vulnerable populations [434]. These ethical implications of AMR also include issues of equity in access to treatments and the responsibilities of stakeholders in antibiotic stewardship [283]. Balancing the urgent need for new therapies against the necessity for rigorous testing and ethical considerations is crucial for the responsible advancement of phage therapy and other innovative interventions in the fight against AMR.

### 9.4. Strategic Roadmap and Challenges Ahead

#### 9.4.1. Strengthening Global Surveillance and Data Sharing

AMR surveillance is crucial for global health, particularly in LMICs, where the burden of bacterial infections is high [364]. However, LMICs face significant challenges in implementing effective AMR surveillance due to limited resources, a weak laboratory capacity, and poor health systems [166]. The GAP has prompted countries to improve their AMR surveillance capacities, but data from these efforts often lack representativeness and standardization [364]. Estimating the global burden of AMR is challenging due to insufficient high-quality, patient-level data, especially in resource-limited settings [435]. To address these issues, a multidisciplinary approach involving various stakeholders is necessary [136]. Strengthening global surveillance networks, improving data sharing, and investing in infrastructure and capacity building are essential for informed decision making and timely interventions against AMR [136].

#### 9.4.2. Integrating AMR into National and Global Health Agendas

Integrating AMR into broader health agendas and the Sustainable Development Goals (SDGs) framework is essential in addressing this challenge [436]. However, current AMR initiatives have had limited impacts, compelling a shift towards emphasizing AMR as being interlinked with other global challenges and incorporating upstream interventions into developmental agendas [437]. While global and national stewardship efforts primarily focus on mitigating antibiotic use in the human and animal sectors, the environmental drivers are often overlooked, particularly water-related factors [432]. Addressing AMR requires a multidisciplinary One Health approach acknowledging the connections between humans, animals, and their shared environment [398]. The successful mitigation of AMR will involve collaboration at the individual, community, and national levels to ensure the continued effectiveness of antimicrobials [398].

#### 9.4.3. Fostering Collaboration across Sectors

Addressing AMR requires collaboration across multiple sectors, including healthcare, agriculture, the environment, and industry. However, implementing this approach is challenging, particularly in LMICs [438]. Barriers include the limited policy visibility of zoonotic diseases, conflicting departmental priorities, and insufficient institutional capacities [438]. Effective multisectoral coordination requires the strengthening of national policy frameworks, establishment of governance structures, and improvement of technical working groups [438]. For instance, poor awareness, inadequate regulations, and limited IPC strategies in Nigeria contribute to inappropriate antibiotic use across sectors [439]. Malaysia also faces similar challenges, with gaps in research assessing AMR’s coexistence across sectors and a limited understanding of antimicrobial use behaviors [430]. To operationalize One Health, countries must invest in governance structures, foster community participation, and promote cross-sectoral collaboration while addressing the socioeconomic and behavioral factors influencing antimicrobial use [439,440].

#### 9.4.4. Promoting Public Awareness and Behavioral Change

Public awareness and education are crucial components in AMR management. Recent studies have explored various interventions to improve the AMR awareness and promote behavioral change. Interactive elements, such as games and videos, have shown higher engagement in public education campaigns [441]. However, there is a lack of evidence on the long-term behavioral impacts and sustained change [442]. In LMICs, multi-faceted interventions targeting healthcare professionals have been most effective when combining education with supportive measures [442]. Researchers emphasize the need for a multidisciplinary approach, incorporating insights from the social sciences, such as education and psychology, to develop more effective AMR interventions [31]. While some interventions have demonstrated cost-effectiveness, more economic evaluations are needed to support decision making in resource-constrained environments [442].

## 10. Conclusions

Antimicrobial resistance arises through genetic changes and environmental pressures, enabling microorganisms to survive exposure to drugs designed to inhibit or kill them. This complexity underscores the need for comprehensive strategies, including prudent antimicrobial use, robust infection control, and ongoing research on novel therapies. The epidemiology of AMR reveals its widespread and increasing prevalence across different pathogens, regions, and sectors, highlighting the urgent need for a coordinated global response. Without concerted action, the world risks a future where common infections become untreatable, severely undermining modern medicine’s achievements.

AMR has grave consequences for global health, leading to higher morbidity, mortality, and healthcare costs and straining economic resources. Addressing this crisis demands a multifaceted effort involving governments, healthcare providers, the pharmaceutical industry, and the public. By promoting ASPs, enhancing IPC, and investing in research and transboundary collaboration, the global community can mitigate the impact of AMR and preserve life-saving treatments for future generations.

The One Health approach is responsible for a paradigm shift in addressing AMR by recognizing the interconnectedness of human, animal, and environmental health. This comprehensive framework supports effective surveillance, coordinated policies, innovative research, and educational initiatives.

Policy and regulatory initiatives are essential in guiding the global AMR response. National and international policies provide frameworks for coordinated action, while regulatory frameworks ensure the responsible use of antimicrobials. Successfully implementing these initiatives requires the overcoming of challenges such as resource limitations, political will, and global coordination. However, with innovation and collaboration, significant progress can be made in combating AMR.

Antimicrobial resistance presents a complex, multifaceted challenge that requires a coordinated global response. The emergence of multidrug-resistant pathogens and novel resistance mechanisms highlights the need for new antimicrobial agents and alternative treatment strategies. The societal and ethical implications of AMR interventions must be carefully considered to ensure the equitable distribution of the benefits. Strengthening global surveillance, integrating AMR into broader health agendas, fostering cross-sectoral collaboration, and promoting public awareness are all vital components of the fight against AMR. By embracing a comprehensive and collaborative approach, we can safeguard the efficacy of antimicrobials and protect global public health for future generations.

As previously stated, the fight against AMR is a defining challenge of our time, requiring a unified global effort that spans disciplines, sectors, and borders. By understanding the mechanisms of resistance, addressing its epidemiological complexities, and embracing innovative approaches through the One Health framework, we can develop sustainable solutions to this growing threat. A collaborative approach that integrates policy, regulation, research, and public engagement is essential to preserving antimicrobials’ effectiveness and ensuring future generations’ health and safety. The time to act is now, and the stakes could not be higher.

## Figures and Tables

**Table 1:** Overview of the main antimicrobial resistance mechanisms found in bacteria, viruses, fungi, and parasites.

Organism	Mechanism	Details	Example	Ref.
**Bacteria**	Target Modification	Antibiotic’s molecular target alteration inhibits drug binding	rRNA mutations hinder aminoglycoside binding	[34,35,36]
	Efflux Pumps	Membrane proteins expel antibiotics and reduce their intracellular concentrations	Efflux of tetracyclines, fluoroquinolones, and macrolides by specific bacterial pumps	[37,38,39]
	Enzymatic Degradation	Enzymes chemically modify or degrade antibiotics and neutralize their effects	β-lactamases hydrolyze the β-lactam ring (penicillin, cephalosporin)	[40,41,42,43]
	Membrane Permeability	Outer membrane permeability reduction limits antibiotic entry	Gram-negative bacteria alter outer membrane proteins to resist drug influx	[44,45,46]
	Biofilm Formation	Biofilms limit antibiotic penetration and enhance resistance	Biofilm-associated resistance in *P. aeruginosa* infections	[47,48,49,50]
**Viruses**	Mutation	Mutations in viral genes encoding enzymes reduce the binding affinity of antiviral drugs	HIV resistance due to mutations in reverse transcriptase and protease	[51,52]
	Changes in Viral Entry Proteins	Surface protein mutations prevent antiviral drugs from achieving host cell entry	Resistance to entry inhibitors in HIV treatment	[53]
	Altered Drug Activation	Mutations impair the activation of antiviral drugs within the virus or host cells	Acyclovir resistance in herpes simplex virus due to *TK* gene mutations	[54]
**Fungi**	Target Modification	Antifungal drugs’ molecular target alteration reduces drug efficacy	Azole resistance in *Candida* spp. due to *ERG11* gene mutations	[55]
	Efflux Pumps	Membrane proteins expel antifungal drugs and reduce their intracellular concentrations	Azole resistance in *C. albicans* due to ABC transporter overexpression	[56,57,58,59,60,61]
	Cell Wall Alteration	Cell wall composition modifications decrease drug binding or penetration	Echinocandin resistance in *Candida* spp. due to *FKS* gene mutations	[62,63]
	Biofilm Formation	Biofilms protect cells from antifungal drugs and difficult infection treatment	Biofilm-related resistance in *Candida* infections associated with medical devices	[64,65,66]
**Parasites**	Target Modification	Parasites acquire mutations in genes encoding drug targets, leading to reduced drug efficacy	Antifolate resistance in *P. falciparum* due to *DHFR* gene mutations	[67,68,69,70,71]
	Efflux pumps	Membrane proteins actively remove drugs from cells and reduce their intracellular concentrations	Chloroquine resistance in *P. falciparum* due to transporter protein expression	[70]
	Metabolic Pathway Alteration	Metabolic pathway alterations to bypass drug effects	Glycolytic pathway alterations confer resistance to drugs in trypanosomes	[72]
	Reduced Drug Activation	Mutations reduce the activation of drugs within parasites, leading to resistance	Metronidazole resistance in *G. lamblia* due to *NTR* mutations	[73,74,75]

**Table 2:** Novel approaches to combat antimicrobial resistance.

Approach	Description	Advantages	Challenges	Examples	Ref.
New Antibiotics with Novel Mechanisms	Development of antibiotics targeting non-traditional bacterial processes.	Target novel aspects of bacterial physiology, useful for resistant bacteria.	Difficulty in the discovery and development of novel classes.	Teixobactin—targets bacterial cell wall synthesis in a new way, with no observed resistance yet.	[324]
Bacteriophage Therapy	Utilizes viruses that specifically infect and kill bacteria.	High specificity, ecological safety, and potential beyond clinical applications.	Large-scale production, stability, and bacterial resistance to phages.	Phage therapy for *P. aeruginosa* infections in cystic fibrosis patients.	[325,326]
Phage-Based Small Molecule Development	Exploits phage–bacteria interactions to identify new antibacterial targets and inhibitory proteins.	Novel targets for drug development, potentially more specific and effective treatments.	Development and scaling challenges.	Phage-derived enzymes (endolysins) are used to treat *S. aureus* infections.	[327]
Antimicrobial Peptides (AMPs)	Naturally occurring peptides with broad-spectrum antibacterial, antiviral, and antifungal properties.	Broad-spectrum activity, lower risk of resistance development, several FDA-approved drugs.	Requires more research on mechanisms of action and clinical optimization.	Daptomycin is used to treat infections by resistant Gram-positive bacteria (e.g., MRSA).	[328,329,330]
Immunotherapies	Monoclonal antibodies target and neutralize bacterial toxins and virulence factors.	High specificity, lower risk of resistance development.	High production costs and the need for targeted pathogen identification.	Bezlotoxumab used to neutralize *C. difficile* toxins.	[331,332]
Probiotics and Fecal Transplant Therapy	Enhances human microbiota to prevent infections.	Restores healthy microbiomes, potentially reducing antibiotic use.	Efficacy varies and needs more standardized research.	FMT used to treat recurrent *C. difficile* infections.	[333]
Pathogen-Oriented Therapy (POT)	Targeted approach using antibiotic conjugates, nanotechnologies, and CRISPR-Cas systems.	Highly specific to pathogens, avoids non-targeted antimicrobial use.	Complex development, regulatory challenges, and scaling issues.	CRISPR-Cas antimicrobials targeting resistant bacteria, antibiotic–antibody conjugates.	[334,335]
Combination Therapies	Use of multiple drugs to prevent or overcome resistance.	Enhances efficacy and prevents resistance development.	Risk of drug interactions and complex treatment regimens.	Ceftazidime-avibactam is used to treat multidrug-resistant Gram-negative bacteria.	[336]
Repurposing Existing Drugs	Using known drugs for new antibacterial purposes.	Cost-effective, faster clinical adoption due to existing safety profiles.	Limited applicability, potential to develop resistance to repurposed drugs.	Colistin is used to treat MDR *Acinetobacter* and *Pseudomonas* species.	[336]
Nanotechnology	Use of nanoparticles to enhance antibiotic efficacy or act as antimicrobial agents.	Enhances antibiotic activity and overcomes biofilm-related resistance.	Safety, bioavailability, and large-scale production.	Silver nanoparticles used in combination with antibiotics to enhance their efficacy.	[337,338]
Artificial Intelligence	AI-driven approaches for drug discovery, resistance prediction, and treatment optimization.	Accelerates drug discovery, predicts resistance, optimizes antimicrobial regimens.	Data quality, model accuracy, and integration into clinical workflows.	IBM’s AI-driven drug discovery used to identify new antibiotics like halicin.	[339,340]
One Health Approach	Multisectoral approach linking human, animal, and environmental health to combat AMR.	Promotes cross-sector collaboration and addresses complex AMR drivers globally.	Requires significant international cooperation and systemic change across multiple industries.	WHO Global Action Plan on AMR, One Health AMR Surveillance Programs (WHO, FAO, OIE).	[341,342,343,344]
